# Supplementary material for: Singlet oxygen-induced signalling depends on the metabolic status of the Chlamydomonas reinhardtii cell
Source: Commun Biol. 2023 May 16;6:529. doi: 10.1038/s42003-023-04872-5 (PMC10188600; doi:10.1038/s42003-023-04872-5)
Supplement: Supplementary file 1 — Supplementary Information [file 42003_2023_4872_MOESM1_ESM.pdf]

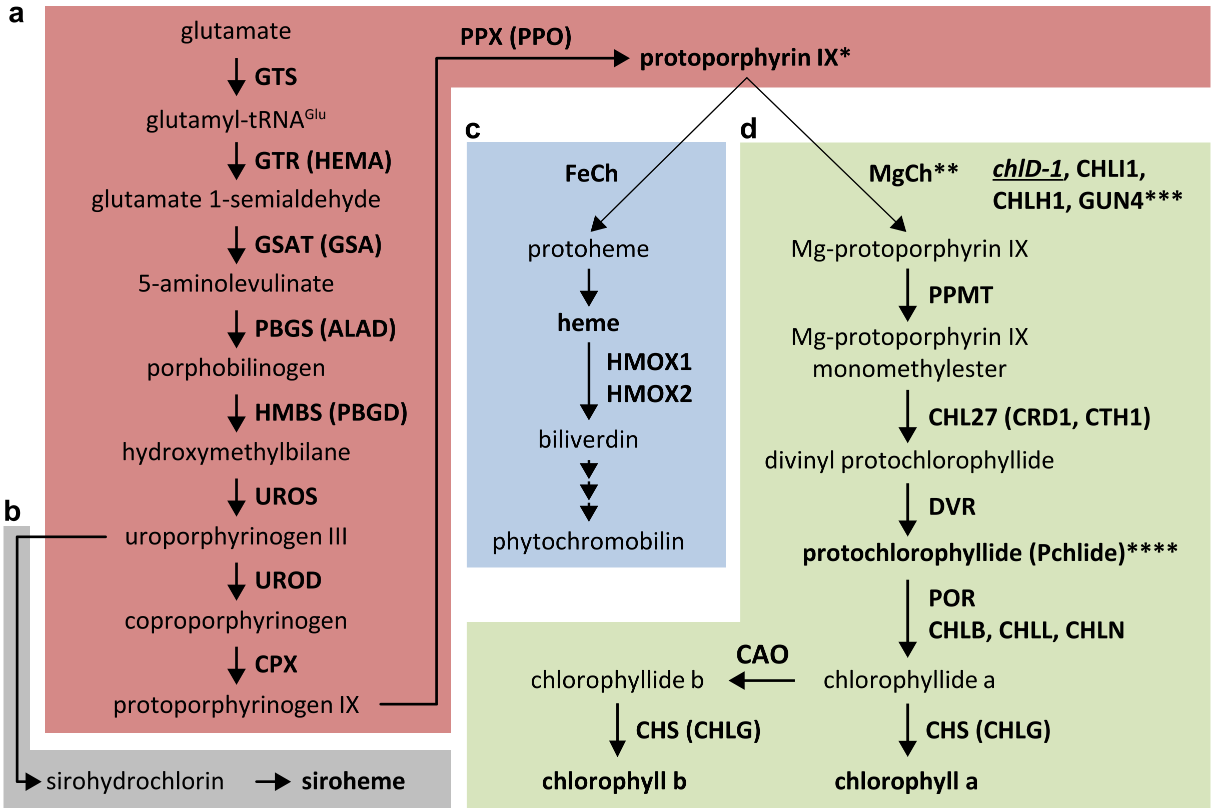

Supplementary Fig. 1 Schematic representation of tetrapyrrole biosynthesis pathway. **a** Common steps from glutamate to protoporphyrin IX (Proto); Proto is marked by an asterisk. **b** The siroheme biosynthesis branch. **c** Heme biosynthesis branch, from ferrochelatase (FeCh) to heme. Subsequent steps of heme catabolism from biliverdin to formation of phytochromobilin are not shown in detail. **d** Steps from Mg-chelatase (MgCh) to chlorophyll formation. MgCh responsible for inserting Mg^{2+} into Proto is marked by a double asterisk. GUN4 protein involved in MgCh function is marked by a triple asterisk. Protochlorophyllide (Pchlide) accumuating in the *flu* mutant of *A. thaliana* is marked by a quadruple asterisk. Mutation in one of the subunits of MgCh, *chlD-1* is underlined.

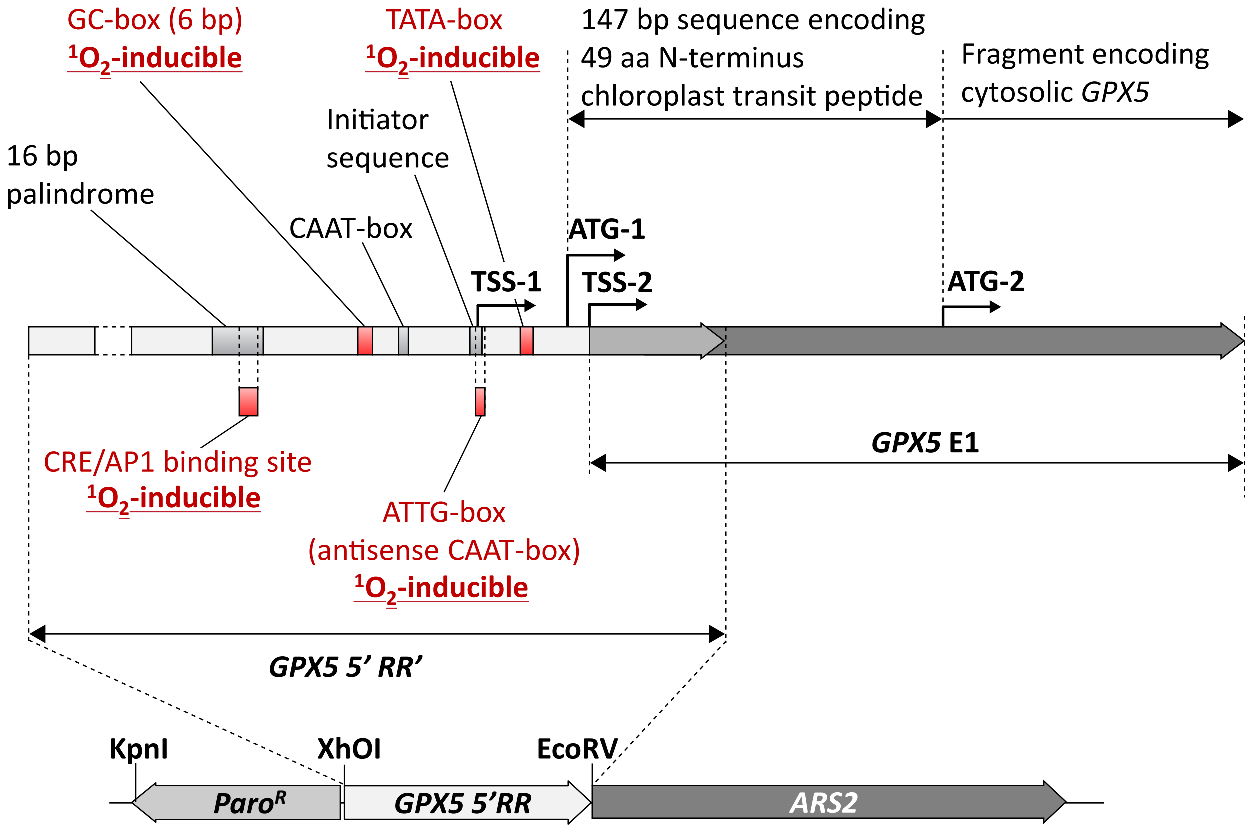

Supplementary Fig. 2 Schematic representation of the *GPX5-ARS2* reporter gene construct. Upper panel, the *GPX5* gene encodes two proteins, one targeted to the chloroplast equipped with the chloroplast transit peptide ($GPX5_{cp}$), and the cytosolic one ($GPX5_{cyt}$). Alternative transcription (TSS) and translation start sites (ATG) are indicated. The $^1O_2$-inducible *cis*-elements in *GPX5* regulatory region (*GPX5'RR*) are indicated, based on Fischer et al.[1]. Lower panel, fusion of *GPX5'RR* with *ARS2*, and the paromomycin resistance cassette ($Paro^R$) in reverse orientation is indicated.

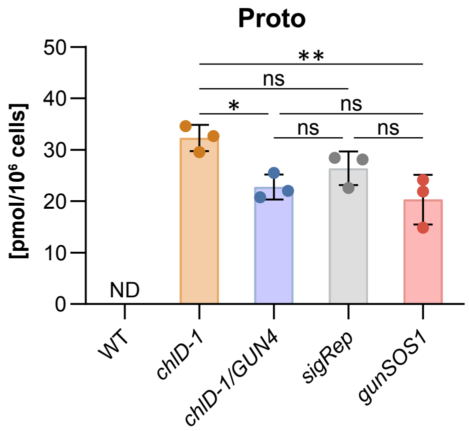

Supplementary Fig. 3 Analysis of Proto content in *gunSOS1*. Mutant impaired in $^{1}O_2$-signaling showed similar Proto accumulation in light compared to *sigRep*; ND, not detectable. Experiments were performed in biological replications (n = 3); the error bars represent calculated ±SD. Significant differences were calculated using one-way ANOVA, pair-wise comparison with the Tukey's post-hoc test, non-significant (ns), *P < 0.05 and **P < 0.01. All mutants unable to synthesize chlorophyll showed significant accumulation of Proto compared to WT (P < 0.0001, not shown).

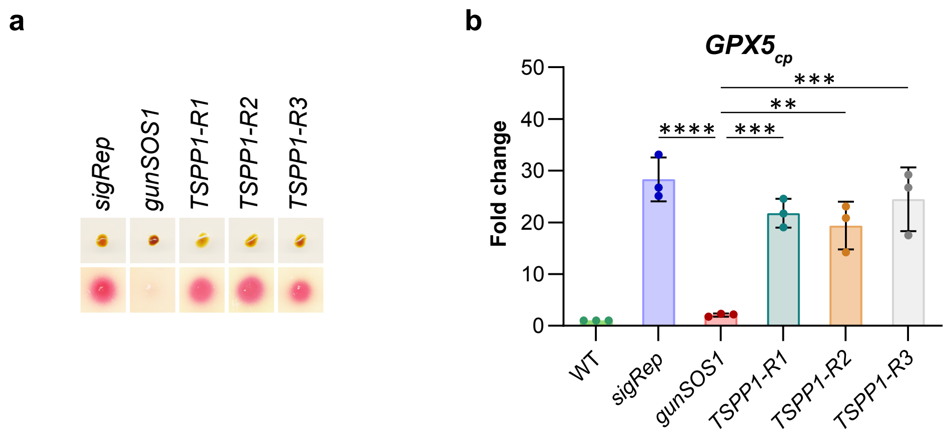

Supplementary Fig. 4 Rescue of the $^1O_2$-signalling in *gunSOS1* by introduction of the wild-type *TSPP1*. **a** Arylsulfatase assay showed higher activity of ARS2 in rescued strains, *TSPP1-R1*, *-R2*, and *-R3* compared to *gunSOS1*. **b** Expression of $GPX5_{cp}$ in the rescued strain compared to *gunSOS1* and *sigRep*. Results are presented as a fold change normalized to WT ($2^{-\Delta\Delta Ct}$, WT = 1); experiments were performed in biological replications ($n = 3$); the error bars represent calculated ±SD. Significant differences were calculated using one-way ANOVA, pair-wise comparison with the Tukey's post-hoc test (non-significant not shown), $^{**}P < 0.01$, $^{***}P < 0.001$, and $^{****}P < 0.0001$. Statistical comparison between mutants and WT are not shown for clarity.

Supplementary Table 1. Metabolites analyzed in *gunSOS1* compared to *sigRep*, and WT (LC-MS/MS). Names and annotations are based on the MetaboAnalyst5.0 portal (https://www.metaboanalyst.ca); The Human Metabolome Database (HMDB), Kyoto Encyclopedia of Genes and Genomes (KEGG).

**Sugars**		
**Match**	**HMDB**	**KEGG**
Trehalose 6-phosphate	HMDB0001124	C00689
Trehalose	HMDB0000975	C01083
D-Glucose	HMDB0000122	C00031
D-Fructose	HMDB0000660	C02336
D-Maltose	HMDB0000163	C00208
Maltotriose	HMDB0001262	C01835
ADP-glucose	HMDB0006557	C00498
Galactose 1-phosphate	HMDB0000645	C00446
Fructose 1-phosphate	HMDB0001076	C01094
Alpha-D-Glucose 1,6-bisphosphate	HMDB0003514	C01231
Glucose 1-phosphate	HMDB0001586	C00446
Fructose 6-phosphate	HMDB0000124	C00085
Mannose 6-phosphate	HMDB0001078	C00275
Fructose 1,6-bisphosphate	HMDB0001058	C00354
Glucose 6-phosphate	HMDB0001401	C00092
**Sugar alcohols and non-sugar metabolites**		
**Match**	**HMDB**	**KEGG**
Sorbitol	HMDB0000247	C00794
myo-Inositol	HMDB0000211	C00137
Glycerol 3-phosphate	HMDB0000126	C00093
Phosphoenolpyruvic acid	HMDB0000263	C00074
cis-Aconitic acid	HMDB0000072	C00417
Isocitric acid	HMDB0000193	C00311
Oxoglutaric acid	HMDB0000208	C00026
Pyruvic acid	HMDB0000243	C00022
Succinic acid	HMDB0000254	C00042
3-Phosphoglyceric acid	HMDB0000807	C00597
Glyceric acid	HMDB0000139	C00258
Citric acid	HMDB0000094	C00158
L-Malic acid	HMDB0000156	C00149
Fumaric acid	HMDB0000134	C00122

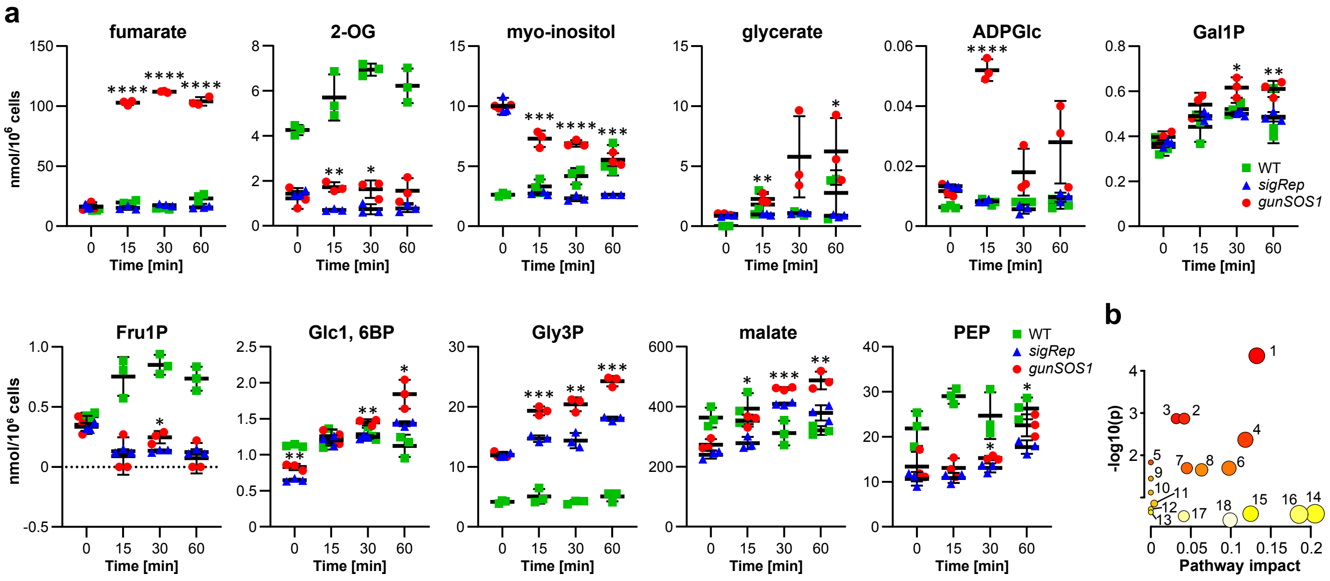

Supplementary Fig. 5 Metabolites with significantly increased content in *gunSOS1* compared to *sigRep* in the light. a LC-MS/MS analysis; fumaric acid (fumarate), oxoglutaric acid (2-OG), myo-inositol, glyceric acid (glycerate), ADP-glucose (ADPGlc), galactose 1-phosphate (Gal1P), fructose 1-phosphate (Fru1P), alpha-D-glucose 1,6-bisphosphate (Glc1, 6BP), glycerol 3-phosphate (Gly3P), L-malic acid (malate), and phosphoenolpyruvic acid (PEP). Measurements were performed in biological triplicates (n = 3), horizontal bars represent the calculated mean, vertical error bars represent calculated ±SD; significant differences were calculated comparing *gunSOS1* to *sigRep* using two-tailed Student's t-test and are indicated by asterisks (non-significant not shown), *$P < 0.05$, **$P < 0.01$, ***$P < 0.001$, and ****$P < 0.0001$. The metabolites for photosynthetic WT are shown as a reference. **b** Impact of accumulating metabolites on cell metabolism analysed using MetaboAnalyst 5.0 (https://www.metaboanalyst.ca), sorted by the P value; 1. tricarboxylic acid cycle (TCA cycle; P = 4.41E-05), 2. pyruvate metabolism (P = 0.00136), 3. starch and sucrose metabolism (P = 0.00136), 4. glyoxylate and dicarboxylate metabolism (P = 0.00429), 5. arginine biosynthesis (P = 0.01473), 6. alanine, aspartate and glutamate metabolism (P = 0.02019), 7. glycerolipid metabolism (P = 0.02019), 8. carbon fixation in photosynthetic organisms (P = 0.02217), 9. galactose metabolism (P = 0.03566), 10. amino sugar and nucleotide sugar metabolism (P = 0.07616), 11. fructose and mannose metabolism (P = 0.13874), 12. tyrosine metabolism (P = 0.1872), 13. phenylalanine, tyrosine and tryptophan biosynthesis (P = 0.22414), 14. inositol phosphate metabolism (P = 0.24202), 15. glycolysis/gluconeogenesis (P = 0.24202), 16. phosphatidylinositol signaling system (P = 0.25082), 17. glycine, serine and threonine metabolism (P = 0.27665), 18. glycerophospholipid metabolism (P = 0.34157); detailed results are presented in Supplementary Table 2.

Supplementary Table 2. Impact of accumulating metabolites on cell metabolism. Details for the analysis performed using MetaboAnalyst 5.0 portal (https://www.metaboanalyst.ca), presented in Supplementary Fig. 5b; sorted by the *P* value.

Pathway name	Total	Expected	Hits	*P*	-log(*P*)	Holm *P*	FDR	Impact
Citrate cycle (TCA cycle)	20	0.22727	4	4.41×10^{-5}	4.3558	0.0037	0.0037	0.132
Pyruvate metabolism	21	0.23864	3	0.00136	2.8674	0.11263	0.038	0.042
Starch and sucrose metabolism	21	0.23864	3	0.00136	2.8674	0.11263	0.038	0.032
Glyoxylate and dicarboxylate metabolism	31	0.35227	3	0.00429	2.3674	0.34764	0.09013	0.118
Arginine biosynthesis	17	0.19318	2	0.01473	1.8317	1	0.2328	0
Alanine, aspartate and glutamate metabolism	20	0.22727	2	0.02019	1.6949	1	0.2328	0.098
Glycerolipid metabolism	20	0.22727	2	0.02019	1.6949	1	0.2328	0.045
Carbon fixation in photosynthetic organisms	21	0.23864	2	0.02217	1.6542	1	0.2328	0.063
Galactose metabolism	27	0.30682	2	0.03566	1.4478	1	0.33281	0
Amino sugar and nucleotide sugar metabolism	41	0.46591	2	0.07616	1.1183	1	0.63976	0
Fructose and mannose metabolism	13	0.14773	1	0.13874	0.85779	1	1	0.004
Tyrosine metabolism	18	0.20455	1	0.1872	0.7277	1	1	0
Phenylalanine, tyrosine and tryptophan biosynthesis	22	0.25	1	0.22414	0.64948	1	1	0
Inositol phosphate metabolism	24	0.27273	1	0.24202	0.61615	1	1	0.205
Glycolysis / Gluconeogenesis	24	0.27273	1	0.24202	0.61615	1	1	0.125
Phosphatidylinositol signaling system	25	0.28409	1	0.25082	0.60064	1	1	0.185
Glycine, serine and threonine metabolism	28	0.31818	1	0.27665	0.55807	1	1	0.041
Glycerophospholipid metabolism	36	0.40909	1	0.34157	0.46652	1	1	0.099

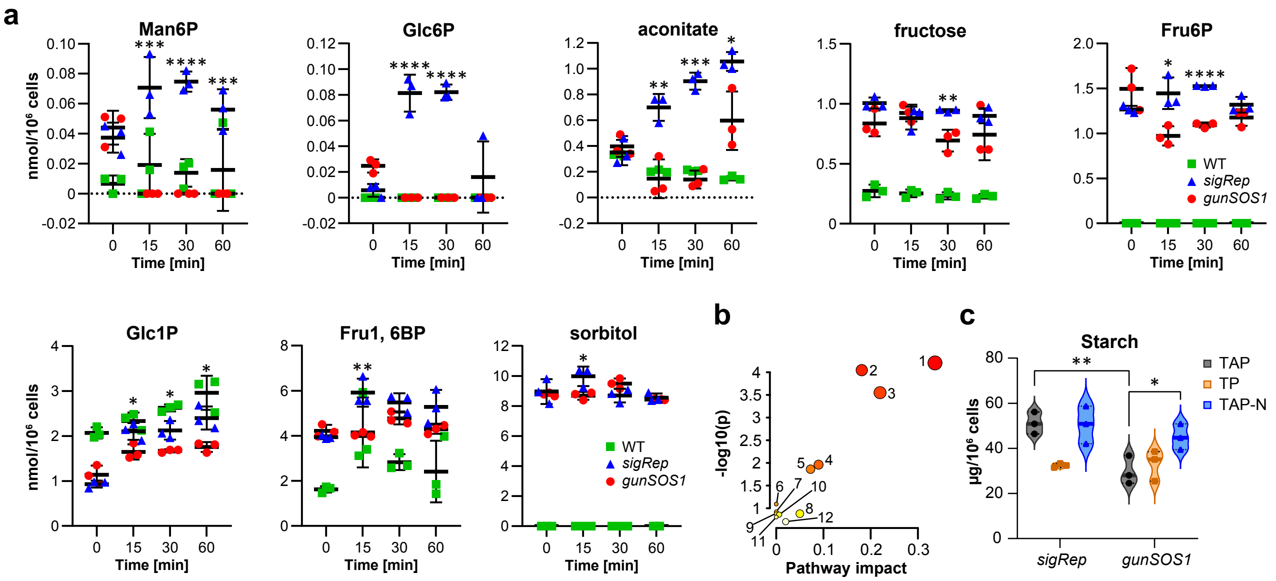

Supplementary Fig. 6 Metabolites with significantly decreased content in *gunSOS1* compared to *sigRep* in the light. a LC-MS/MS analysis; mannose 6-phosphate (Man6P), glucose 6-phosphate (Glc6P), cis-aconitic acid (aconitate), D-fructose (fructose), fructose 6-phosphate (Fru6P), glucose 1-phosphate (Glc1P), fructose 1,6-bisphosphate (Fru1, 6BP), and sorbitol. Measurements were performed in biological triplicates (n = 3), horizontal bars represent the calculated mean, vertical error bars represent calculated ±SD; significant differences were calculated comparing *gunSOS1* to *sigRep* using two-tailed Student's t-test and are indicated by asterisks (non-significant not shown), *$P < 0.05$, **$P < 0.01$, ***$P < 0.001$, and ****$P < 0.0001$. The metabolites for photosynthetic WT are shown as a reference. **b** Impact of deficient metabolites on cell metabolism analysed using MetaboAnalyst 5.0 (https://www.metaboanalyst.ca), sorted by the P value; 1. fructose and mannose metabolism (P = 6.23E-05), 2. amino sugar and nucleotide sugar metabolism (P = 8.99E-05), 3. starch and sucrose metabolism (P = 0.000282), 4. Glycolysis / Gluconeogenesis (P = 0.01094), 5. Galactose metabolism (P = 0.013767), 6. pentose and glucuronate interconversions (P = 0.081139), 7. pentose phosphate pathway (P = 0.1195), 8. tricarboxylic acid cycle (TCA cycle; P = 0.13197), 9. glycerolipid metabolism (P = 0.13197), 10. carbon fixation in photosynthetic organisms (P = 0.13815), 11. inositol phosphate metabolism (P = 0.15646), 12. glyoxylate and dicarboxylate metabolism (P = 0.19785); detailed results are presented in Supplemental Table 3. **c** Starch accumulation in *gunSOS1* compared to *sigRep*. Measurements were performed in biological triplicates (n = 3); median is shown as a center line; upper and lower quartiles are shown as dotted lines. Significant differences were calculated using two-tailed Student's t-test and are indicated by asterisks (non-significant not shown), *$P < 0.05$, **$P < 0.01$.

Supplementary Table 3. Impact of deficient metabolites on cell metabolism. Details for the analysis performed using MetaboAnalyst 5.0 portal (https://www.metaboanalyst.ca), presented in Supplementary Fig. 6b; sorted by the *P* value.

**Pathway name**	**Total**	**Expected**	**Hits**	***P***	**-log(*P*)**	**Holm *P***	**FDR**	**Impact**
Fructose and mannose metabolism	13	0.090909	3	6.23×10^{-5}	4.2058	0.00523	0.003775	0.336
Amino sugar and nucleotide sugar metabolism	41	0.28671	4	8.99×10^{-5}	4.0463	0.007461	0.003775	0.181
Starch and sucrose metabolism	21	0.14685	3	0.000282	3.5498	0.023122	0.007895	0.22
Glycolysis / Gluconeogenesis	24	0.16783	2	0.01094	1.961	0.88616	0.22974	0.09
Galactose metabolism	27	0.18881	2	0.013767	1.8612	1	0.23128	0.073
Pentose and glucuronate interconversions	12	0.083916	1	0.081139	1.0908	1	1	0
Pentose phosphate pathway	18	0.12587	1	0.1195	0.92262	1	1	0
Citrate cycle (TCA cycle)	20	0.13986	1	0.13197	0.87951	1	1	0.05
Glycerolipid metabolism	20	0.13986	1	0.13197	0.87951	1	1	0
Carbon fixation in photosynthetic organisms	21	0.14685	1	0.13815	0.85964	1	1	0.006
Inositol phosphate metabolism	24	0.16783	1	0.15646	0.8056	1	1	0
Glyoxylate and dicarboxylate metabolism	31	0.21678	1	0.19785	0.70366	1	1	0.02

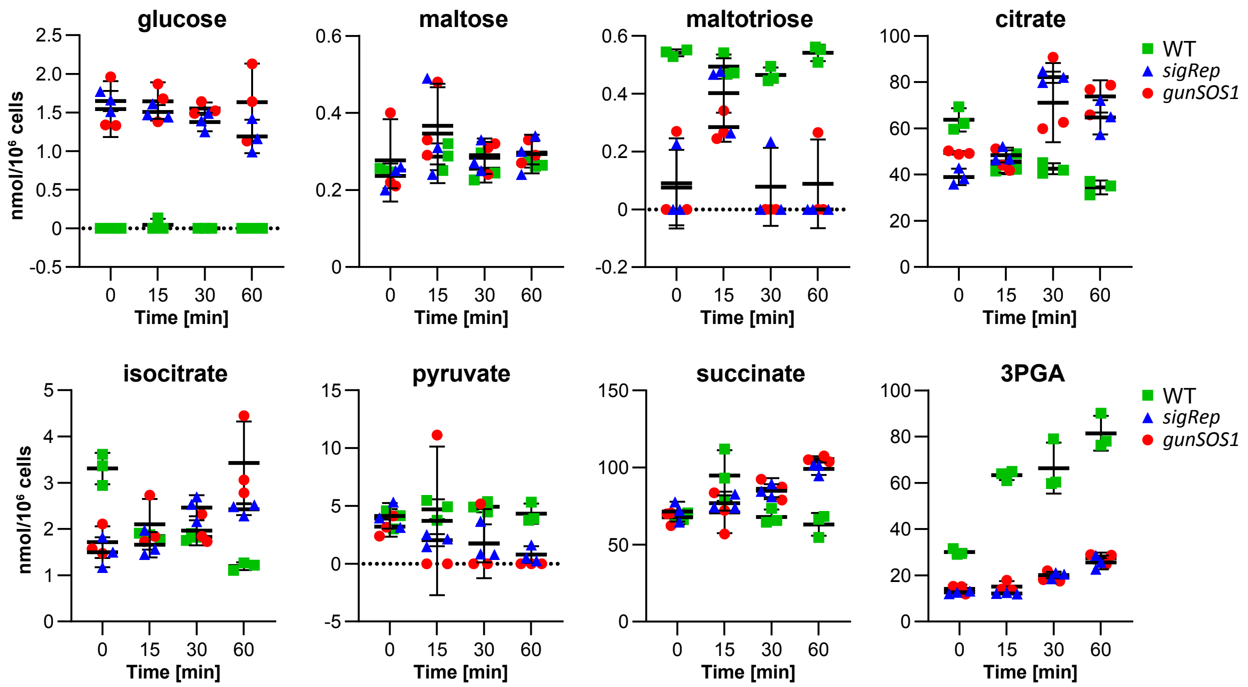

Supplementary Fig. 7 Metabolites content in *gunSOS1* compared to *sigRep*. D-glucose (glucose), D-maltose (maltose), maltotriose, citric acid (citrate), isocitric acid (isocitrate), pyruvic acid (pyruvate), succinic acid (succinate), 3-phosphoglyceric acid (3PGA). Measurements were performed in biological triplicates and are presented as mean (n = 3), horizontal bars represent the calculated mean, vertical error bars represent calculated ±SD; significant differences were calculated comparing *gunSOS1* to *sigRep* using two-tailed Student's *t*-test. Concerning non-photosynthetic strains exposed to $^1O_2$-stress, metabolites presented here did not show significant change in light. The measurement of metabolites content in photosynthetic WT is shown only as a reference.

a

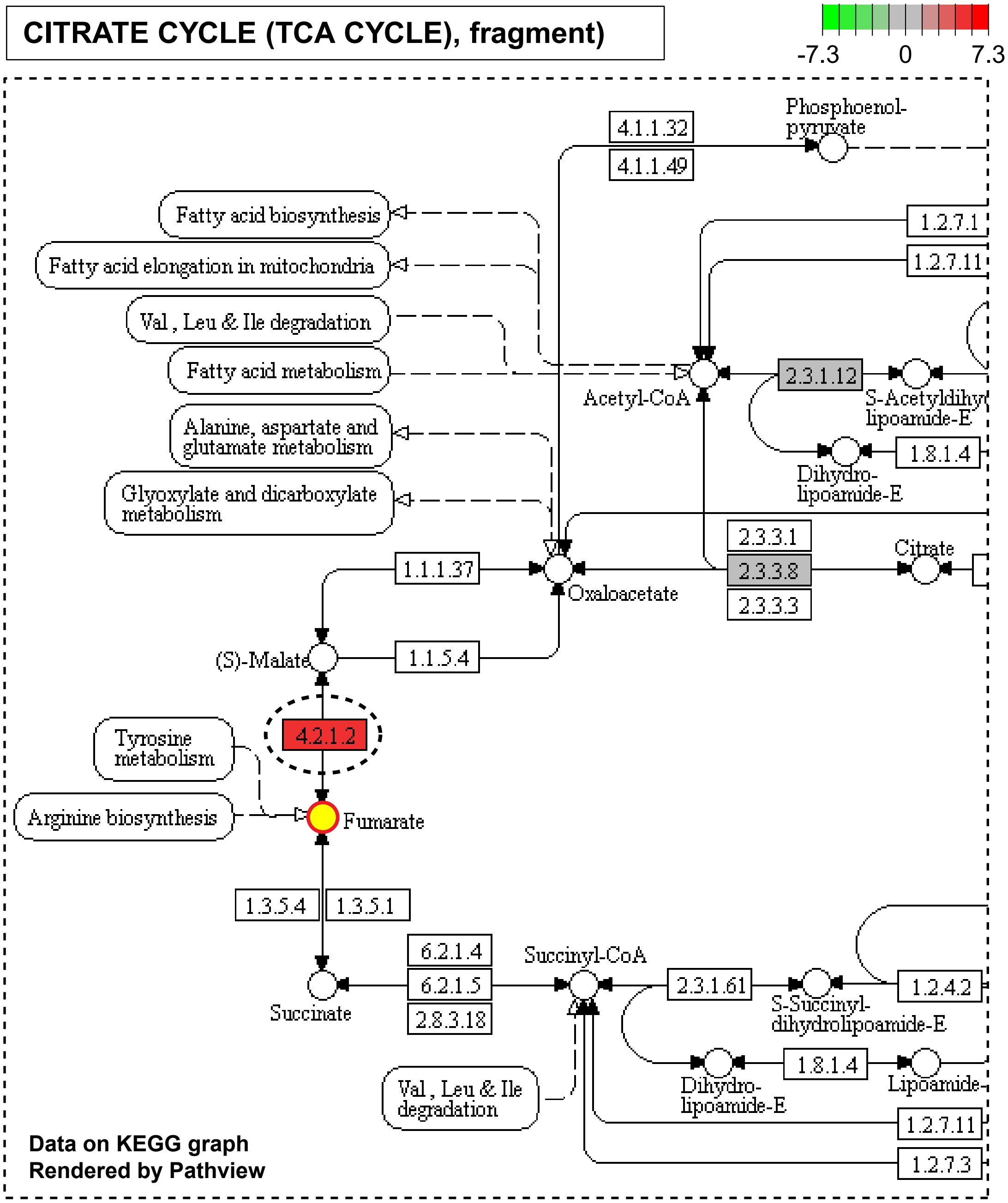
CITRATE CYCLE (TCA CYCLE), fragment)
-7.3
0
7.3
4.1.1.32
4.1.1.49
Phosphoenol-pyruvate
Fatty acid biosynthesis
Fatty acid elongation in mitochondria
Val , Leu & Ile degradation
Fatty acid metabolism
1.2.7.1
1.2.7.11
Acetyl-CoA
2.3.1.12
S-Acetyldihy
lipoamide-E
1.8.1.4
Dihydro-lipoamide-E
Alanine, aspartate and glutamate metabolism
Glyoxylate and dicarboxylate metabolism
2.3.3.1
2.3.3.8
2.3.3.3
Citrate
1.1.1.37
Oxaloacetate
(S)-Malate
1.1.5.4
4.2.1.2
Tyrosine metabolism
Arginine biosynthesis
Fumarate
1.3.5.4
1.3.5.1
6.2.1.4
6.2.1.5
2.8.3.18
Succinyl-CoA
2.3.1.61
S-Succinyl-dihydrolipoamide-E
1.2.4.2
Succinate
1.8.1.4
Dihydro-lipoamide-E
Lipoamide-
Val , Leu & Ile degradation
1.2.7.11
1.2.7.3
Data on KEGG graph
Rendered by Pathview

b

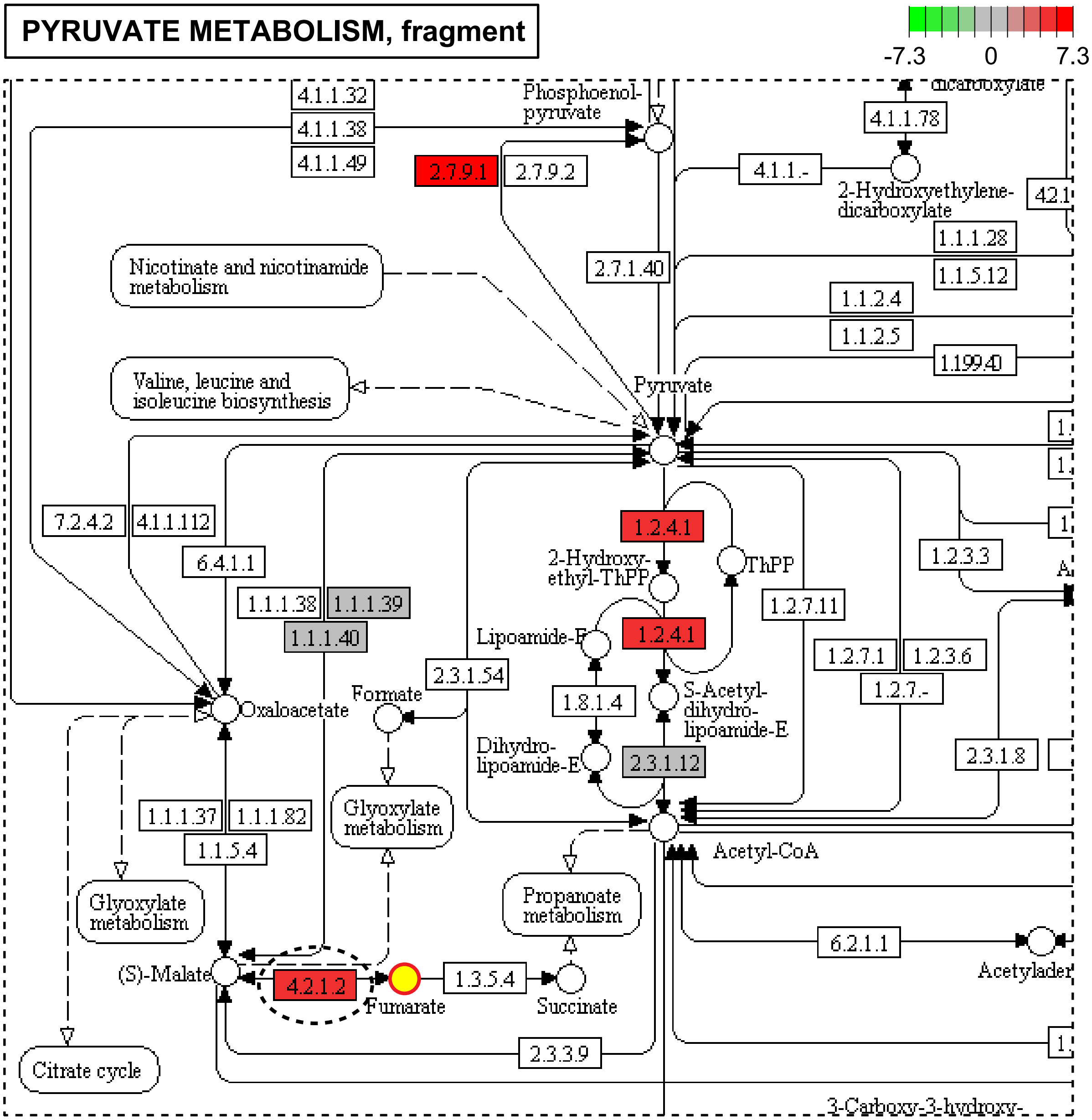

Data on KEGG graph
Rendered by Pathview

c

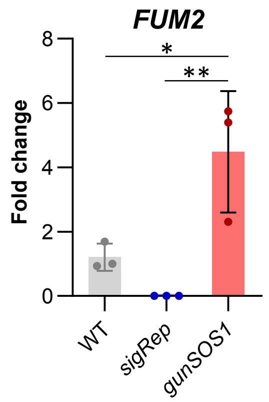

d

EC 4.2.1.2	FUM1/FUM2
Name	fumarate hydratase;<br>fumarase;<br>L-malate hydro-lyase;<br>(S)-malate hydro-lyase
Class	Lyases;<br>Carbon-oxygen lyases;<br>Hydro-lyases
Sysname	(S)-malate hydro-lyase (fumarate-forming)
Reaction(IUBMB)	(S)-malate = fumarate + H_2O [RN:R01082]
Reaction(KEGG)	R01082
Substrate	(S)-malate [CPD:C00149]
Product	**fumarate** [CPD:C00122];<br>H_2O [CPD:C00001]

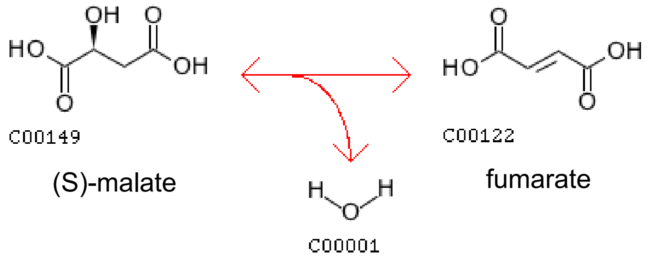

Supplementary Fig. 8 Accumulation of fumarate in *gunSOS1* compared to *sigRep* is explained by increased expression of *FUMARATE HYDRATASE 2*. a Fragment of the citrate cycle (TCA cycle) with indicated fumarate. **b** Fragment of the pyruvate metabolism with indicated fumarate. The RNA-seq results showed 5.17-fold increase in *FUMARATE HYDRATASE 2* (*FUM2*, Cre01.g020223.v5.5, EC 4.2.1.2, marked with dashed-line oval) expression in *gunSOS1* compared to *sigRep*. **c** qRT-PCR showed nearly 4 times higher *FUM2* transcript in *gunSOS1* compared to WT, while it was not detectable in *sigRep*. Results are presented as as a fold change ($2^{-\Delta\Delta Ct}$) normalized to the mean of Ct_{exp}-Ct_{ref} of WT; experiment was performed in biological replications (n = 3); the error bars represent calculated ±SD. Significant differences were calculated using one-way ANOVA, pair-wise comparison with the Tukey's post-hoc test (non-significant not shown), *P < 0.05, **P < 0.01. **d** FUM2 (EC 4.2.1.2) catalyses hydrolysis of malate to fumarate and H_2O, based on KEGG analysis.

a

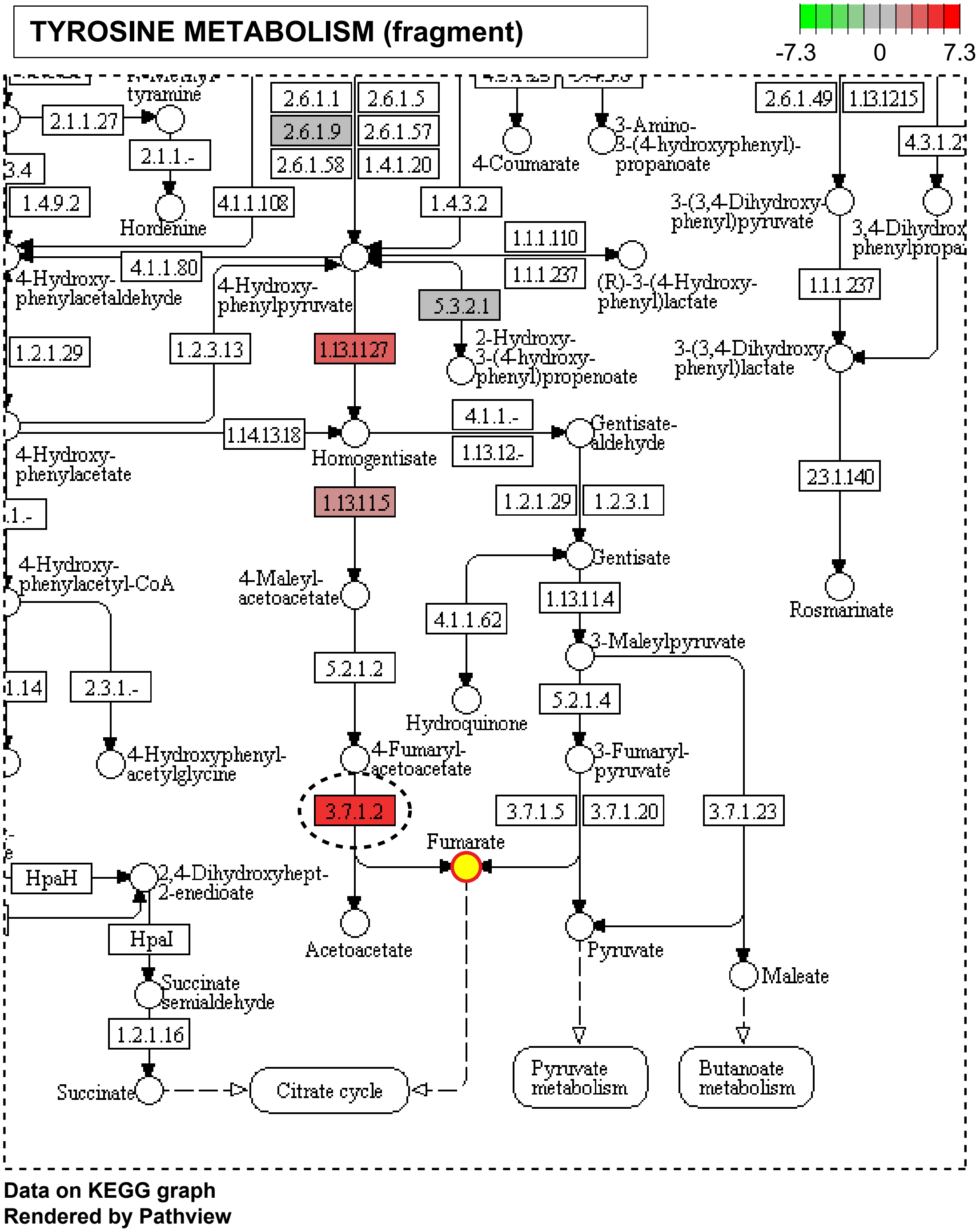
TYROSINE METABOLISM (fragment)
-7.3
0
7.3
tyramine
2.1.1.27
2.1.1.-
3.4
1.4.9.2
Hordenine
4.1.1.108
2.6.1.1
2.6.1.5
2.6.1.9
2.6.1.57
2.6.1.58
1.4.1.20
1.4.3.2
4-Coumarate
3-Amino-
3-(4-hydroxyphenyl)-
propanoate
2.6.1.49
1.13.12.15
4.3.1.2
3-(3,4-Dihydroxy-
phenyl)pyruvate
3,4-Dihydrox
phenylpropa
4.1.1.80
4-Hydroxy-
phenylacetaldehyde
4-Hydroxy-
phenylpyruvate
1.1.1.110
1.1.1.237
(R)-3-(4-Hydroxy-
phenyl)lactate
5.3.2.1
1.1.1.237
1.2.1.29
1.2.3.13
1.13.11.27
2-Hydroxy-
3-(4-hydroxy-
phenyl)propenoate
3-(3,4-Dihydroxy-
phenyl)lactate
1.14.13.18
Homogentisate
4.1.1.-
1.13.12.-
Gentisate-
aldehyde
4-Hydroxy-
phenylacetate
1.13.11.5
1.2.1.29
1.2.3.1
2.3.1.140
.1.-
Gentisate
4-Hydroxy-
phenylacetyl-CoA
4-Maleyl-
acetoacetate
1.13.11.4
4.1.1.62
Rosmarinate
3-Maleylpyruvate
5.2.1.2
1.14
2.3.1.-
5.2.1.4
Hydroquinone
4-Hydroxyphenyl-
acetylglycine
4-Fumaryl-
acetoacetate
3-Fumaryl-
pyruvate
3.7.1.2
3.7.1.5
3.7.1.20
3.7.1.23
Fumarate
HpaH
2,4-Dihydroxyhept-
2-enedioate
HpaI
Acetoacetate
Pyruvate
Maleate
Succinate
semialdehyde
1.2.1.16
Succinate
Citrate cycle
Pyruvate
metabolism
Butanoate
metabolism
Data on KEGG graph
Rendered by Pathview

b

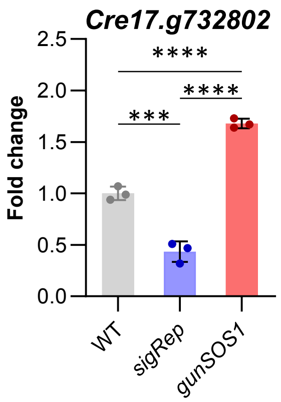

c

EC 3.7.1.2	Cre17.g732802.v5.5
Name	fumarylacetoacetase; beta-diketonase; fumarylacetoacetate hydrolase
Class	Acting on carbon-carbon bonds; In ketonic substances
Sysname	4-fumarylacetoacetate fumarylhydrolase
Reaction(IUBMB)	4-fumarylacetoacetate + H_2O = acetoacetate + fumarate [RN:R01364]
Reaction(KEGG)	R01364
Substrate	4-fumarylacetoacetate [CPD:C01061]; H_2O [CPD:C00001]
Product	acetoacetate [CPD:C00164];<br>fumarate [CPD:C00122]

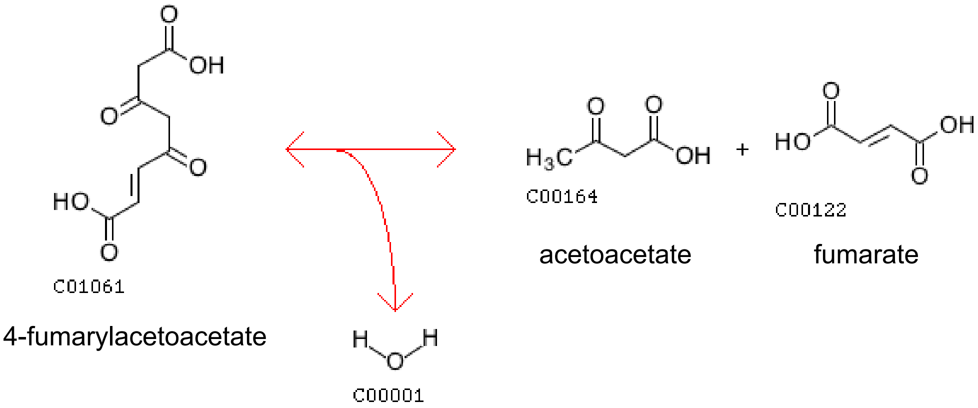

Supplementary Fig. 9 Accumulation of fumarate in *gunSOS1* compared to *sigRep* is explained by increased expression of *FUMARYLACETOACETASE*. a Fragment of the tyrosine metabolism with indicated fumarate, based on KEGG, modified. The RNA-seq results showed 4.71-fold increase in *FUMARYLACETOACETASE* (Cre17.g732802, EC 3.7.1.2, marked with dashed-line oval) expression in *gunSOS1* compared to *sigRep*. **b** qRT-PCR showed nearly 4-fold increase in *FUMARYLACETOACETASE* (Cre17.g732802) transcript in *gunSOS1* compared to *sigRep*. Results are presented as as a fold change ($2^{-\Delta\Delta Ct}$) normalized to the mean of Ct_{exp}-Ct_{ref} of WT; experiment was performed in biological replications (n = 3); the error bars represent calculated ±SD. Significant differences were calculated using one-way ANOVA, pair-wise comparison with the Tukey's post-hoc test, ***P < 0.001 and ****P < 0.0001. **c** Fumarylacetoacetase (EC 3.7.1.2) catalyses hydrolysis of fumarylacetoacetate producing acetoacetate, fumarate, and H_2O, based on KEGG analysis.

a

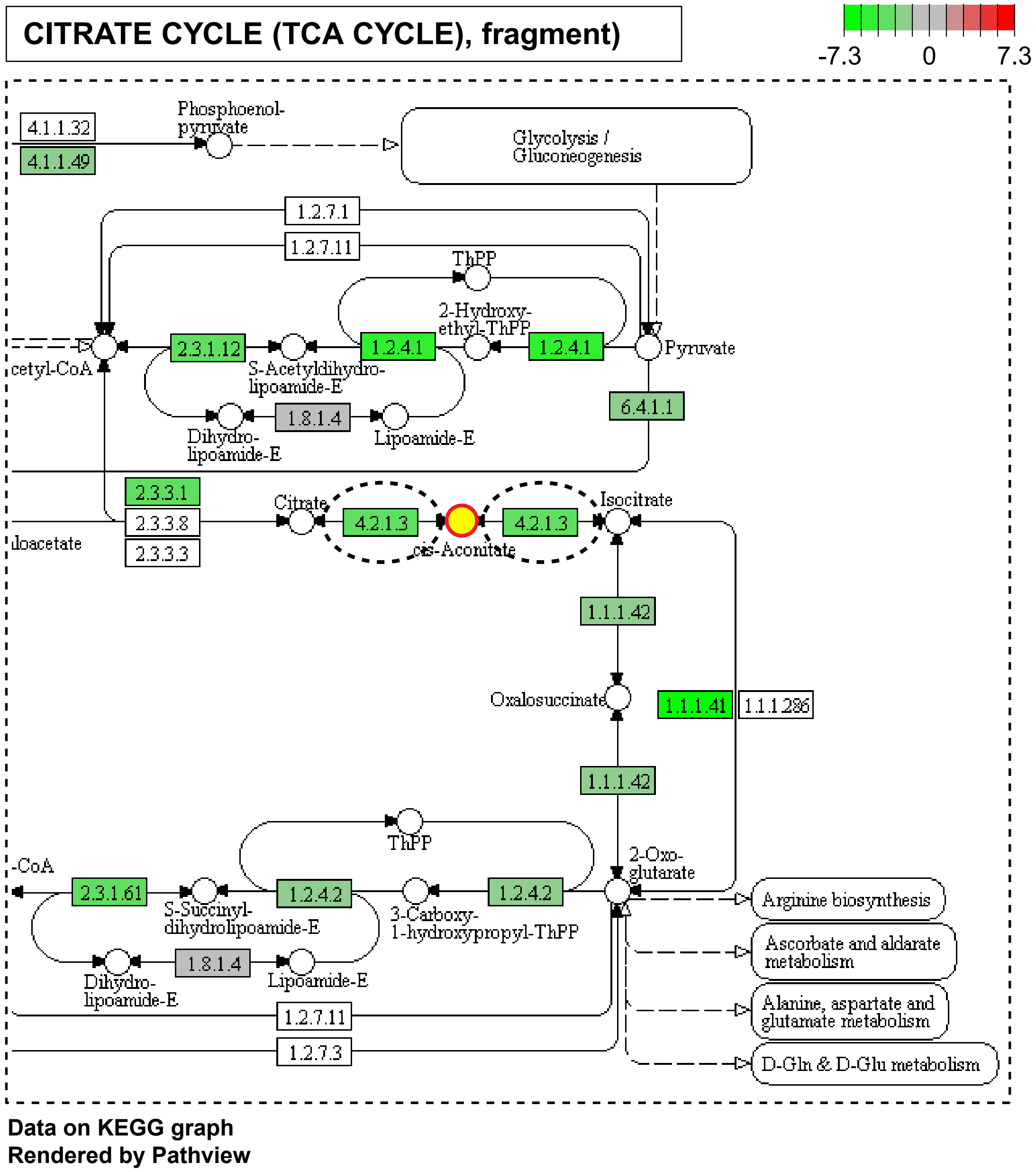
CITRATE CYCLE (TCA CYCLE), fragment)
-7.3
0
7.3
4.1.1.32
4.1.1.49
Phosphoenol-
pyruvate
Glycolysis /
Gluconeogenesis
1.2.7.1
1.2.7.11
ThPP
2-Hydroxy-
ethyl-ThPP
2.3.1.12
1.2.4.1
1.2.4.1
Pyruvate
cetyl-CoA
S-Acetyldihydro-
lipoamide-E
1.8.1.4
Dihydro-
lipoamide-E
Lipoamide-E
6.4.1.1
2.3.3.1
2.3.3.8
2.3.3.3
loacetate
Citrate
4.2.1.3
4.2.1.3
cis-Aconitate
Isocitrate
1.1.1.42
Oxalosuccinate
1.1.1.41
1.1.1.286
1.1.1.42
ThPP
-CoA
2.3.1.61
S-Succinyl-
dihydrolipoamide-E
1.2.4.2
3-Carboxy-
1-hydroxypropyl-ThPP
1.2.4.2
2-Oxo-
glutarate
1.8.1.4
Dihydro-
lipoamide-E
Lipoamide-E
1.2.7.11
1.2.7.3
Arginine biosynthesis
Ascorbate and aldarate
metabolism
Alanine, aspartate and
glutamate metabolism
D-Gln & D-Glu metabolism
Data on KEGG graph
Rendered by Pathview

b

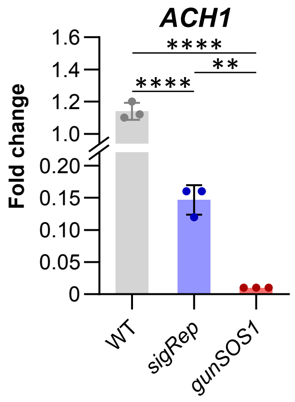
ACH1
Fold change
1.6
1.4
1.2
1.0
0.20
0.15
0.10
0.05
0
****
**
****
WT
sigRep
gunSOS1

c

EC 4.2.1.3	ACH1
Name	aconitate hydratase; cis-aconitase; aconitase; AcnB; 2-methylaconitate hydratase; citrate(isocitrate) hydro-lyase
Class	Lyases; Carbon-oxygen lyases; Hydro-lyases
Sysname	citrate(isocitrate) hydro-lyase (cis-aconitate-forming)
Reaction(IUBMB)	citrate = isocitrate (overall reaction) [RN:R01324];<br>(1a) citrate = cis-aconitate + H_2O [RN:R01325];<br>(1b) cis-aconitate + H_2O = isocitrate [RN:R01900]
Reaction(KEGG)	R01324 R01325 R01900
Substrate	citrate [CPD:C00158]; cis-aconitate [CPD:C00417]; H_2O [CPD:C00001]
Product	isocitrate [CPD:C00311]; cis-aconitate [CPD:C00417]; H_2O [CPD:C00001]
Comment	Besides interconverting citrate and cis-aconitate, it also interconverts cis-aconitate with isocitrate and, hence, interconverts citrate and isocitrate. The equilibrium mixture is 91% citrate, 6% isocitrate and 3% aconitate. cis-aconitate is used to designate the isomer (Z)-prop-1-ene-1,2,3-tricarboxylate. An iron-sulfur protein, containing a [4Fe-4S] cluster to which the substrate binds.

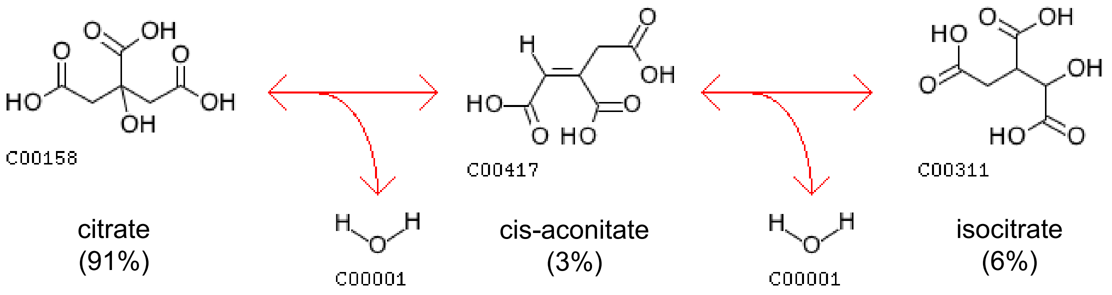

Supplementary Fig. 10 Deficiency in aconitate in *gunSOS1* compared to *sigRep* is explained by decreased expression of *ACONITATE HYDRATASE 1*. a Fragment of the TCA cycle with indicated aconitate, based on KEGG, modified. The RNA-seq results showed 2.81-fold decrease in *ACONITATE HYDRATASE 1* (*ACH1*; Cre01.g042750; EC 4.2.1.3) expression in *gunSOS1* compared to *sigRep*. **b** Based on qRT-PCR, *ACH1* transcript level was nearly 15 times lower in *gunSOS1* compared to *sigRep*. Results are presented as as a fold change ($2^{-\Delta\Delta Ct}$) normalized to the mean of Ct_{exp}-Ct_{ref} of WT; experiment was performed in biological replications (n = 3); the error bars represent calculated ±SD. Significant differences were calculated using one-way ANOVA, pair-wise comparison with the Tukey's post-hoc test, **P < 0.01, ****P < 0.0001. **c** Catalytic reaction of ACH1 (EC 4.2.1.3), based on KEGG analysis.

Supplementary Table 4. Statistical analyses for *GPX5*$_{cyt}$ expression following treatment with fumarate, presented in Fig. 6a. *P*, probability (significance test); *SS*, sum of squares; *DF*, degree of freedom; *MS*, means square; *F*, F-value (Fisher test); *DFn*, degrees of freedom in the numerator; *DFd*, degrees of freedom in the denominator; *SE*, standard error of the sample mean; *N*, sample number.

**Two-way ANOVA Ordinary**					
Alpha	0.05				
**Source of Variation**	**% of total variation**	**P value**	**P value summary**	**Significant?**	
Interaction	27.86	<0.0001	****	Yes	
strain	54.47	<0.0001	****	Yes	
metabolite	15.36	<0.0001	****	Yes	
**ANOVA table**	**SS**	**DF**	**MS**	**F (DFn, DFd)**	**P value**
Interaction	120.7	6	20.11	F (6, 24) = 48.21	P<0.0001
strain	236.0	2	118.0	F (2, 24) = 282.8	P<0.0001
metabolite	66.51	3	22.17	F (3, 24) = 53.15	P<0.0001
Residual	10.01	24	0.4172		
**Data summary**					
Number of columns (metabolite)	4				
Number of rows (strain)	3				
Number of values	36				
**Two-way ANOVA Multiple comparison**					
Number of families	3				
Number of comparisons per family	3				
Alpha	0.05				

**Dunnett's multiple comparisons test**	**Mean Diff.**	**95.00% CI of diff.**	**Below threshold?**	**Summary**	**Adjusted P Value**
WT					
control vs. 20 µM Fum	0.7367	-0.5854 to 2.059	No	ns	0.3810
control vs. 50 µM Fum	0.6733	-0.6487 to 1.995	No	ns	0.4509
control vs. 100 µM Fum	0.6733	-0.6487 to 1.995	No	ns	0.4509
sigRep					
control vs. 20 µM Fum	4.690	3.368 to 6.012	Yes	****	<0.0001
control vs. 50 µM Fum	7.970	6.648 to 9.292	Yes	****	<0.0001
control vs. 100 µM Fum	10.53	9.211 to 11.86	Yes	****	<0.0001
gunSOS1					
control vs. 20 µM Fum	-0.06667	-1.389 to 1.255	No	ns	0.9985
control vs. 50 µM Fum	-0.2433	-1.565 to 1.079	No	ns	0.9387
control vs. 100 µM Fum	-0.2600	-1.582 to 1.062	No	ns	0.9270

**Test details**	**Mean 1**	**Mean 2**	**Mean Diff.**	**SE of diff.**	**N1**	**N2**	**q**	**DF**
WT								
control vs. 20 µM Fum	1.000	0.2633	0.7367	0.5274	3	3	1.397	24.00
control vs. 50 µM Fum	1.000	0.3267	0.6733	0.5274	3	3	1.277	24.00
control vs. 100 µM Fum	1.000	0.3267	0.6733	0.5274	3	3	1.277	24.00
sigRep								
control vs. 20 µM Fum	11.59	6.903	4.690	0.5274	3	3	8.893	24.00
control vs. 50 µM Fum	11.59	3.623	7.970	0.5274	3	3	15.11	24.00
control vs. 100 µM Fum	11.59	1.060	10.53	0.5274	3	3	19.97	24.00
gunSOS1								
control vs. 20 µM Fum	0.1133	0.1800	-0.06667	0.5274	3	3	0.1264	24.00
control vs. 50 µM Fum	0.1133	0.3567	-0.2433	0.5274	3	3	0.4614	24.00
control vs. 100 µM Fum	0.1133	0.3733	-0.2600	0.5274	3	3	0.4930	24.00

Supplementary Table 5. Statistical analyses for *GPX5*$_{cp}$ expression following treatment with fumarate, presented in Fig. 6a. *P*, probability (significance test); *SS*, sum of squares; *DF*, degree of freedom; *MS*, means square; *F*, F-value (Fisher test); *DFn*, degrees of freedom in the numerator; *DFd*, degrees of freedom in the denominator; *SE*, standard error of the sample mean; *N*, sample number.

**Two-way ANOVA Ordinary**					
Alpha	0.05				
**Source of Variation**	**% of total variation**	**P value**	**P value summary**	**Significant?**	
Interaction	34.57	<0.0001	****	Yes	
strain	40.98	<0.0001	****	Yes	
metabolite	23.29	<0.0001	****	Yes	
**ANOVA table**	**SS**	**DF**	**MS**	**F (DFn, DFd)**	**P value**
Interaction	19.99	6	3.332	F (6, 24) = 118.9	P<0.0001
strain	23.69	2	11.85	F (2, 24) = 422.6	P<0.0001
metabolite	13.47	3	4.489	F (3, 24) = 160.1	P<0.0001
Residual	0.6727	24	0.02803		

**Data summary**	
Number of columns (metabolite)	4
Number of rows (strain)	3
Number of values	36
**Two-way ANOVA Multiple comparison**	
Number of families	3
Number of comparisons per family	3
Alpha	0.05

**Dunnett's multiple comparisons test**	**Mean Diff.**	**95.00% CI of diff.**	**Below threshold?**	**Summary**	**Adjusted P Value**
WT					
control vs. 20 µM Fum	0.7500	0.4073 to 1.093	Yes	****	<0.0001
control vs. 50 µM Fum	0.6767	0.3340 to 1.019	Yes	***	0.0001
control vs. 100 µM Fum	0.7033	0.3606 to 1.046	Yes	****	<0.0001
sigRep					
control vs. 20 µM Fum	1.353	1.011 to 1.696	Yes	****	<0.0001
control vs. 50 µM Fum	3.690	3.347 to 4.033	Yes	****	<0.0001
control vs. 100 µM Fum	3.930	3.587 to 4.273	Yes	****	<0.0001
gunSOS1					
control vs. 20 µM Fum	-0.02333	-0.3660 to 0.3194	No	ns	0.9965
control vs. 50 µM Fum	-0.1033	-0.4460 to 0.2394	No	ns	0.7919
control vs. 100 µM Fum	-0.1033	-0.4460 to 0.2394	No	ns	0.7919

**Test details**	**Mean 1**	**Mean 2**	**Mean Diff.**	**SE of diff.**	**N1**	**N2**	**q**	**DF**
WT								
control vs. 20 µM Fum	1.000	0.2500	0.7500	0.1367	3	3	5.486	24.00
control vs. 50 µM Fum	1.000	0.3233	0.6767	0.1367	3	3	4.950	24.00
control vs. 100 µM Fum	1.000	0.2967	0.7033	0.1367	3	3	5.145	24.00
sigRep								
control vs. 20 µM Fum	4.223	2.870	1.353	0.1367	3	3	9.900	24.00
control vs. 50 µM Fum	4.223	0.5333	3.690	0.1367	3	3	26.99	24.00
control vs. 100 µM Fum	4.223	0.2933	3.930	0.1367	3	3	28.75	24.00
gunSOS1								
control vs. 20 µM Fum	0.05000	0.07333	-0.02333	0.1367	3	3	0.1707	24.00
control vs. 50 µM Fum	0.05000	0.1533	-0.1033	0.1367	3	3	0.7559	24.00
control vs. 100 µM Fum	0.05000	0.1533	-0.1033	0.1367	3	3	0.7559	24.00

Supplementary Table 6. Statistical analyses for *GPX5*$_{cyt}$ expression following treatment with 2-oxoglutarate (2-OG), presented in Fig. 6b. *P*, probability (significance test); *SS*, sum of squares; *DF*, degree of freedom; *MS*, means square; *F*, F-value (Fisher test); *DFn*, degrees of freedom in the numerator; *DFd*, degrees of freedom in the denominator; *SE*, standard error of the sample mean; *N*, sample number.

**Two-way ANOVA Ordinary**					
Alpha	0.05				
**Source of Variation**	**% of total variation**	**P value**	**P value summary**	**Significant?**	
Interaction	24.72	<0.0001	****	Yes	
strain	63.03	<0.0001	****	Yes	
metabolite	10.39	<0.0001	****	Yes	
**ANOVA table**	**SS**	**DF**	**MS**	**F (DFn, DFd)**	**P value**
Interaction	476.1	6	79.35	F (6, 24) = 53.09	P<0.0001
strain	1214	2	607.1	F (2, 24) = 406.2	P<0.0001
metabolite	200.1	3	66.69	F (3, 24) = 44.62	P<0.0001
Residual	35.87	24	1.495		

**Data summary**	
Number of columns (metabolite)	4
Number of rows (strain)	3
Number of values	36
**Two-way ANOVA Multiple comparison**	
Number of families	3
Number of comparisons per family	3
Alpha	0.05

**Dunnett's multiple comparisons test**	**Mean Diff.**	**95.00% CI of diff.**	**Below threshold?**	**Summary**	**Adjusted P Value**
WT					
control vs. 20 µM 2-OG	-0.1567	-2.659 to 2.346	No	ns	0.9972
control vs. 50 µM 2-OG	0.2967	-2.206 to 2.799	No	ns	0.9819
control vs. 100 µM 2-OG	-0.9700	-3.472 to 1.532	No	ns	0.6505
sigRep					
control vs. 20 µM 2-OG	3.963	1.461 to 6.466	Yes	**	0.0016
control vs. 50 µM 2-OG	14.11	11.60 to 16.61	Yes	****	<0.0001
control vs. 100 µM 2-OG	18.58	16.08 to 21.09	Yes	****	<0.0001
gunSOS1					
control vs. 20 µM 2-OG	-0.5067	-3.009 to 1.996	No	ns	0.9212
control vs. 50 µM 2-OG	-0.2833	-2.786 to 2.219	No	ns	0.9842
control vs. 100 µM 2-OG	-0.7933	-3.296 to 1.709	No	ns	0.7675

**Test details**	**Mean 1**	**Mean 2**	**Mean Diff.**	**SE of diff.**	**N1**	**N2**	**q**	**DF**
WT								
control vs. 20 µM 2-OG	1.000	1.157	-0.1567	0.9982	3	3	0.1570	24.00
control vs. 50 µM 2-OG	1.000	0.7033	0.2967	0.9982	3	3	0.2972	24.00
control vs. 100 µM 2-OG	1.000	1.970	-0.9700	0.9982	3	3	0.9718	24.00
sigRep								
control vs. 20 µM 2-OG	22.54	18.58	3.963	0.9982	3	3	3.971	24.00
control vs. 50 µM 2-OG	22.54	8.433	14.11	0.9982	3	3	14.13	24.00
control vs. 100 µM 2-OG	22.54	3.957	18.58	0.9982	3	3	18.62	24.00
gunSOS1								
control vs. 20 µM 2-OG	0.5167	1.023	-0.5067	0.9982	3	3	0.5076	24.00
control vs. 50 µM 2-OG	0.5167	0.8000	-0.2833	0.9982	3	3	0.2838	24.00
control vs. 100 µM 2-OG	0.5167	1.310	-0.7933	0.9982	3	3	0.7948	24.00

Supplementary Table 7. Statistical analyses for $GPX5_{cp}$ expression following treatment with 2-oxoglutarate (2-OG), presented in Fig. 6b. *P*, probability (significance test); *SS*, sum of squares; *DF*, degree of freedom; *MS*, means square; *F*, F-value (Fisher test); *DFn*, degrees of freedom in the numerator; *DFd*, degrees of freedom in the denominator; *SE*, standard error of the sample mean; *N*, sample number.

**Two-way ANOVA Ordinary**					
Alpha	0.05				
**Source of Variation**	**% of total variation**	**P value**	**P value summary**	**Significant?**	
Interaction	34.40	<0.0001	****	Yes	
strain	51.49	<0.0001	****	Yes	
metabolite	12.28	<0.0001	****	Yes	
**ANOVA table**	**SS**	**DF**	**MS**	**F (DFn, DFd)**	**P value**
Interaction	105.9	6	17.65	F (6, 24) = 75.20	P<0.0001
strain	158.5	2	79.25	F (2, 24) = 337.7	P<0.0001
metabolite	37.79	3	12.60	F (3, 24) = 53.68	P<0.0001
Residual	5.632	24	0.2347		
**Data summary**					
Number of columns (metabolite)	4				
Number of rows (strain)	3				
Number of values	36				
**Two-way ANOVA Multiple comparison**					
Number of families	3				
Number of comparisons per family	3				
Alpha	0.05				

**Dunnett's multiple comparisons test**	**Mean Diff.**	**95.00% CI of diff.**	**Below threshold?**	**Summary**	**Adjusted P Value**
WT					
control vs. 20 µM 2-OG	-0.1833	-1.175 to 0.8082	No	ns	0.9380
control vs. 50 µM 2-OG	0.1533	-0.8382 to 1.145	No	ns	0.9619
control vs. 100 µM 2-OG	-1.403	-2.395 to -0.4118	Yes	**	0.0045
sigRep					
control vs. 20 µM 2-OG	4.050	3.058 to 5.042	Yes	****	<0.0001
control vs. 50 µM 2-OG	7.960	6.968 to 8.952	Yes	****	<0.0001
control vs. 100 µM 2-OG	8.413	7.422 to 9.405	Yes	****	<0.0001
gunSOS1					
control vs. 20 µM 2-OG	-0.2900	-1.282 to 0.7015	No	ns	0.8057
control vs. 50 µM 2-OG	-0.1000	-1.092 to 0.8915	No	ns	0.9887
control vs. 100 µM 2-OG	-0.4233	-1.415 to 0.5682	No	ns	0.5840

**Test details**	**Mean 1**	**Mean 2**	**Mean Diff.**	**SE of diff.**	**N1**	**N2**	**q**	**DF**
WT								
control vs. 20 µM 2-OG	1.000	1.183	-0.1833	0.3955	3	3	0.4635	24.00
control vs. 50 µM 2-OG	1.000	0.8467	0.1533	0.3955	3	3	0.3877	24.00
control vs. 100 µM 2-OG	1.000	2.403	-1.403	0.3955	3	3	3.548	24.00
sigRep								
control vs. 20 µM 2-OG	10.39	6.337	4.050	0.3955	3	3	10.24	24.00
control vs. 50 µM 2-OG	10.39	2.427	7.960	0.3955	3	3	20.12	24.00
control vs. 100 µM 2-OG	10.39	1.973	8.413	0.3955	3	3	21.27	24.00
gunSOS1								
control vs. 20 µM 2-OG	0.2400	0.5300	-0.2900	0.3955	3	3	0.7332	24.00
control vs. 50 µM 2-OG	0.2400	0.3400	-0.1000	0.3955	3	3	0.2528	24.00
control vs. 100 µM 2-OG	0.2400	0.6633	-0.4233	0.3955	3	3	1.070	24.00

Supplementary Table 8. Statistical analyses for $GPX5_{cyt}$ expression following treatment with myo-inositol (myo-Ins), presented in Fig. 6c. *P*, probability (significance test); *SS*, sum of squares; *DF*, degree of freedom; *MS*, means square; *F*, F-value (Fisher test); *DFn*, degrees of freedom in the numerator; *DFd*, degrees of freedom in the denominator; *SE*, standard error of the sample mean; *N*, sample number.

**Two-way ANOVA Ordinary**					
Alpha	0.05				
**Source of Variation**	**% of total variation**	**P value**	**P value summary**	**Significant?**	
Interaction	38.28	<0.0001	****	Yes	
strain	40.82	<0.0001	****	Yes	
metabolite	19.29	<0.0001	****	Yes	
**ANOVA table**	**SS**	**DF**	**MS**	**F (DFn, DFd)**	**P value**
Interaction	146.6	6	24.43	F (6, 24) = 94.78	P<0.0001
strain	156.3	2	78.14	F (2, 24) = 303.2	P<0.0001
metabolite	73.86	3	24.62	F (3, 24) = 95.51	P<0.0001
Residual	6.186	24	0.2578		
**Data summary**					
Number of columns (metabolite)	4				
Number of rows (strain)	3				
Number of values	36				
**Two-way ANOVA Multiple comparison**					
Number of families	3				
Number of comparisons per family	3				
Alpha	0.05				

**Dunnett's multiple comparisons test**	**Mean Diff.**	**95.00% CI of diff.**	**Below threshold?**	**Summary**	**Adjusted P Value**
WT					
control vs. 20 µM myo-Ins	0.2600	-0.7792 to 1.299	No	ns	0.8655
control vs. 50 µM myo-Ins	0.3500	-0.6892 to 1.389	No	ns	0.7357
control vs. 100 µM myo-Ins	0.1800	-0.8592 to 1.219	No	ns	0.9480
sigRep					
control vs. 20 µM myo-Ins	4.357	3.317 to 5.396	Yes	****	<0.0001
control vs. 50 µM myo-Ins	10.24	9.204 to 11.28	Yes	****	<0.0001
control vs. 100 µM myo-Ins	10.09	9.054 to 11.13	Yes	****	<0.0001
gunSOS1					
control vs. 20 µM myo-Ins	-0.1833	-1.223 to 0.8558	No	ns	0.9453
control vs. 50 µM myo-Ins	-0.3500	-1.389 to 0.6892	No	ns	0.7357
control vs. 100 µM myo-Ins	-0.08000	-1.119 to 0.9592	No	ns	0.9949

**Test details**	**Mean 1**	**Mean 2**	**Mean Diff.**	**SE of diff.**	**N1**	**N2**	**q**	**DF**
WT								
control vs. 20 µM myo-Ins	1.000	0.7400	0.2600	0.4145	3	3	0.6272	24.00
control vs. 50 µM myo-Ins	1.000	0.6500	0.3500	0.4145	3	3	0.8443	24.00
control vs. 100 µM myo-Ins	1.000	0.8200	0.1800	0.4145	3	3	0.4342	24.00
sigRep								
control vs. 20 µM myo-Ins	11.34	6.980	4.357	0.4145	3	3	10.51	24.00
control vs. 50 µM myo-Ins	11.34	1.093	10.24	0.4145	3	3	24.71	24.00
control vs. 100 µM myo-Ins	11.34	1.243	10.09	0.4145	3	3	24.35	24.00
gunSOS1								
control vs. 20 µM myo-Ins	0.5333	0.7167	-0.1833	0.4145	3	3	0.4423	24.00
control vs. 50 µM myo-Ins	0.5333	0.8833	-0.3500	0.4145	3	3	0.8443	24.00
control vs. 100 µM myo-Ins	0.5333	0.6133	-0.08000	0.4145	3	3	0.1930	24.00

Supplementary Table 9. Statistical analyses for *GPX5*$_{cp}$ expression following treatment with myo-inositol (myo-Ins), presented in Fig. 6c. *P*, probability (significance test); *SS*, sum of squares; *DF*, degree of freedom; *MS*, means square; *F*, F-value (Fisher test); *DFn*, degrees of freedom in the numerator; *DFd*, degrees of freedom in the denominator; *SE*, standard error of the sample mean; *N*, sample number.

**Two-way ANOVA Ordinary**					
Alpha	0.05				
**Source of Variation**	**% of total variation**	**P value**	**P value summary**	**Significant?**	
Interaction	43.01	<0.0001	****	Yes	
strain	33.37	<0.0001	****	Yes	
metabolite	17.52	<0.0001	****	Yes	
**ANOVA table**	**SS**	**DF**	**MS**	**F (DFn, DFd)**	**P value**
Interaction	33.49	6	5.582	F (6, 24) = 28.20	P<0.0001
strain	25.99	2	12.99	F (2, 24) = 65.64	P<0.0001
metabolite	13.64	3	4.548	F (3, 24) = 22.98	P<0.0001
Residual	4.751	24	0.1979		

**Data summary**	
Number of columns (metabolite)	4
Number of rows (strain)	3
Number of values	36
**Two-way ANOVA Multiple comparison**	
Number of families	3
Number of comparisons per family	3
Alpha	0.05

**Dunnett's multiple comparisons test**	**Mean Diff.**	**95.00% CI of diff.**	**Below threshold?**	**Summary**	**Adjusted P Value**
WT					
control vs. 20 µM myo-Ins	0.1533	-0.7573 to 1.064	No	ns	0.9518
control vs. 50 µM myo-Ins	0.02000	-0.8907 to 0.9307	No	ns	>0.9999
control vs. 100 µM myo-Ins	-0.2233	-1.134 to 0.6873	No	ns	0.8719
sigRep					
control vs. 20 µM myo-Ins	2.123	1.213 to 3.034	Yes	****	<0.0001
control vs. 50 µM myo-Ins	4.727	3.816 to 5.637	Yes	****	<0.0001
control vs. 100 µM myo-Ins	4.700	3.789 to 5.611	Yes	****	<0.0001
gunSOS1					
control vs. 20 µM myo-Ins	-0.1700	-1.081 to 0.7407	No	ns	0.9364
control vs. 50 µM myo-Ins	-0.2933	-1.204 to 0.6173	No	ns	0.7595
control vs. 100 µM myo-Ins	-0.06333	-0.9740 to 0.8473	No	ns	0.9962

**Test details**	**Mean 1**	**Mean 2**	**Mean Diff.**	**SE of diff.**	**N1**	**N2**	**q**	**DF**
WT								
control vs. 20 µM myo-Ins	1.000	0.8467	0.1533	0.3633	3	3	0.4221	24.00
control vs. 50 µM myo-Ins	1.000	0.9800	0.02000	0.3633	3	3	0.05506	24.00
control vs. 100 µM myo-Ins	1.000	1.223	-0.2233	0.3633	3	3	0.6148	24.00
sigRep								
control vs. 20 µM myo-Ins	5.370	3.247	2.123	0.3633	3	3	5.845	24.00
control vs. 50 µM myo-Ins	5.370	0.6433	4.727	0.3633	3	3	13.01	24.00
control vs. 100 µM myo-Ins	5.370	0.6700	4.700	0.3633	3	3	12.94	24.00
gunSOS1								
control vs. 20 µM myo-Ins	0.3400	0.5100	-0.1700	0.3633	3	3	0.4680	24.00
control vs. 50 µM myo-Ins	0.3400	0.6333	-0.2933	0.3633	3	3	0.8075	24.00
control vs. 100 µM myo-Ins	0.3400	0.4033	-0.06333	0.3633	3	3	0.1743	24.00

Supplementary Table 10. Statistical analyses for *GPX5*$_{cyt}$ expression following treatment with mannose 6-phosphate (Man6P), presented in Fig. 6d. *P*, probability (significance test); *SS*, sum of squares; *DF*, degree of freedom; *MS*, means square; *F*, F-value (Fisher test); *DFn*, degrees of freedom in the numerator; *DFd*, degrees of freedom in the denominator; *SE*, standard error of the sample mean; *N*, sample number.

**Two-way ANOVA Ordinary**					
Alpha	0.05				
**Source of Variation**	**% of total variation**	**P value**	**P value summary**	**Significant?**	
Interaction	29.88	<0.0001	****	Yes	
strain	55.04	<0.0001	****	Yes	
metabolite	13.35	<0.0001	****	Yes	
**ANOVA table**	**SS**	**DF**	**MS**	**F (DFn, DFd)**	**P value**
Interaction	2545	6	424.2	F (6, 24) = 69.22	P<0.0001
strain	4687	2	2344	F (2, 24) = 382.5	P<0.0001
metabolite	1137	3	379.1	F (3, 24) = 61.87	P<0.0001
Residual	147.1	24	6.128		

**Data summary**	
Number of columns (metabolite)	4
Number of rows (strain)	3
Number of values	36
**Two-way ANOVA Multiple comparison**	
Number of families	3
Number of comparisons per family	3
Alpha	0.05

**Dunnett's multiple comparisons test**	**Mean Diff.**	**95.00% CI of diff.**	**Below threshold?**	**Summary**	**Adjusted P Value**
WT					
control vs. 20 µM Man6P	-1.050	-6.117 to 4.017	No	ns	0.9163
control vs. 50 µM Man6P	-2.200	-7.267 to 2.867	No	ns	0.5718
control vs. 100 µM Man6P	-2.453	-7.520 to 2.614	No	ns	0.4900
sigRep					
control vs. 20 µM Man6P	-40.39	-45.46 to -35.32	Yes	****	<0.0001
control vs. 50 µM Man6P	-11.24	-16.31 to -6.173	Yes	****	<0.0001
control vs. 100 µM Man6P	4.410	-0.6569 to 9.477	No	ns	0.0976
gunSOS1					
control vs. 20 µM Man6P	-0.1267	-5.194 to 4.940	No	ns	0.9999
control vs. 50 µM Man6P	-0.9000	-5.967 to 4.167	No	ns	0.9443
control vs. 100 µM Man6P	-2.490	-7.557 to 2.577	No	ns	0.4786

**Test details**	**Mean 1**	**Mean 2**	**Mean Diff.**	**SE of diff.**	**N1**	**N2**	**q**	**DF**
WT								
control vs. 20 µM Man6P	1.000	2.050	-1.050	2.021	3	3	0.5195	24.00
control vs. 50 µM Man6P	1.000	3.200	-2.200	2.021	3	3	1.088	24.00
control vs. 100 µM Man6P	1.000	3.453	-2.453	2.021	3	3	1.214	24.00
sigRep								
control vs. 20 µM Man6P	14.83	55.22	-40.39	2.021	3	3	19.98	24.00
control vs. 50 µM Man6P	14.83	26.07	-11.24	2.021	3	3	5.561	24.00
control vs. 100 µM Man6P	14.83	10.42	4.410	2.021	3	3	2.182	24.00
gunSOS1								
control vs. 20 µM Man6P	1.553	1.680	-0.1267	2.021	3	3	0.06267	24.00
control vs. 50 µM Man6P	1.553	2.453	-0.9000	2.021	3	3	0.4453	24.00
control vs. 100 µM Man6P	1.553	4.043	-2.490	2.021	3	3	1.232	24.00

Supplementary Table 11. Statistical analyses for $GPX5_{cp}$ expression following treatment with mannose 6-phosphate (Man6P), presented in Fig. 6d. *P*, probability (significance test); *SS*, sum of squares; *DF*, degree of freedom; *MS*, means square; *F*, F-value (Fisher test); *DFn*, degrees of freedom in the numerator; *DFd*, degrees of freedom in the denominator; *SE*, standard error of the sample mean; *N*, sample number.

**Two-way ANOVA Ordinary**					
Alpha	0.05				
**Source of Variation**	**% of total variation**	**P value**	**P value summary**	**Significant?**	
Interaction	32.12	<0.0001	****	Yes	
strain	51.16	<0.0001	****	Yes	
metabolite	13.87	<0.0001	****	Yes	
**ANOVA table**	**SS**	**DF**	**MS**	**F (DFn, DFd)**	**P value**
Interaction	162.0	6	27.00	F (6, 24) = 45.15	P<0.0001
strain	258.0	2	129.0	F (2, 24) = 215.7	P<0.0001
metabolite	69.95	3	23.32	F (3, 24) = 39.00	P<0.0001
Residual	14.35	24	0.5979		

**Data summary**	
Number of columns (metabolite)	4
Number of rows (strain)	3
Number of values	36

**Two-way ANOVA Multiple comparison**	
Number of families	3
Number of comparisons per family	3
Alpha	0.05

**Dunnett's multiple comparisons test**	**Mean Diff.**	**95.00% CI of diff.**	**Below threshold?**	**Summary**	**Adjusted P Value**
WT					
control vs. 20 µM Man6P	-1.183	-2.766 to 0.3994	No	ns	0.1745
control vs. 50 µM Man6P	-1.983	-3.566 to -0.4006	Yes	*	0.0120
control vs. 100 µM Man6P	-2.273	-3.856 to -0.6906	Yes	**	0.0040
sigRep					
control vs. 20 µM Man6P	-10.14	-11.73 to -8.561	Yes	****	<0.0001
control vs. 50 µM Man6P	-4.030	-5.613 to -2.447	Yes	****	<0.0001
control vs. 100 µM Man6P	0.6100	-0.9727 to 2.193	No	ns	0.6543
gunSOS1					
control vs. 20 µM Man6P	0.2067	-1.376 to 1.789	No	ns	0.9763
control vs. 50 µM Man6P	-0.2433	-1.826 to 1.339	No	ns	0.9625
control vs. 100 µM Man6P	-0.9033	-2.486 to 0.6794	No	ns	0.3625

**Test details**	**Mean 1**	**Mean 2**	**Mean Diff.**	**SE of diff.**	**N1**	**N2**	**q**	**DF**
WT								
control vs. 20 µM Man6P	1.000	2.183	-1.183	0.6314	3	3	1.874	24.00
control vs. 50 µM Man6P	1.000	2.983	-1.983	0.6314	3	3	3.141	24.00
control vs. 100 µM Man6P	1.000	3.273	-2.273	0.6314	3	3	3.601	24.00
sigRep								
control vs. 20 µM Man6P	3.843	13.99	-10.14	0.6314	3	3	16.07	24.00
control vs. 50 µM Man6P	3.843	7.873	-4.030	0.6314	3	3	6.383	24.00
control vs. 100 µM Man6P	3.843	3.233	0.6100	0.6314	3	3	0.9662	24.00
gunSOS1								
control vs. 20 µM Man6P	0.7633	0.5567	0.2067	0.6314	3	3	0.3273	24.00
control vs. 50 µM Man6P	0.7633	1.007	-0.2433	0.6314	3	3	0.3854	24.00
control vs. 100 µM Man6P	0.7633	1.667	-0.9033	0.6314	3	3	1.431	24.00

Supplementary Table 12. Statistical analyses for *GPX5*$_{cyt}$ expression following treatment with glucose 6-phosphate (Glc6P), presented in Fig. 6e. *P*, probability (significance test); *SS*, sum of squares; *DF*, degree of freedom; *MS*, means square; *F*, F-value (Fisher test); *DFn*, degrees of freedom in the numerator; *DFd*, degrees of freedom in the denominator; *SE*, standard error of the sample mean; *N*, sample number.

**Two-way ANOVA Ordinary**					
Alpha	0.05				
**Source of Variation**	**% of total variation**	**P value**	**P value summary**	**Significant?**	
Interaction	8.457	<0.0001	****	Yes	
strain	86.02	<0.0001	****	Yes	
metabolite	3.982	<0.0001	****	Yes	
**ANOVA table**	**SS**	**DF**	**MS**	**F (DFn, DFd)**	**P value**
Interaction	373.6	6	62.26	F (6, 24) = 21.96	P<0.0001
strain	3800	2	1900	F (2, 24) = 670.2	P<0.0001
metabolite	175.9	3	58.64	F (3, 24) = 20.69	P<0.0001
Residual	68.04	24	2.835		
**Data summary**					
Number of columns (metabolite)	4				
Number of rows (strain)	3				
Number of values	36				
**Two-way ANOVA Multiple comparison**					
Number of families	3				
Number of comparisons per family	3				
Alpha	0.05				

**Dunnett's multiple comparisons test**	**Mean Diff.**	**95.00% CI of diff.**	**Below threshold?**	**Summary**	**Adjusted P Value**
WT					
control vs. 20 µM Glc6P	0.03333	-3.413 to 3.480	No	ns	>0.9999
control vs. 50 µM Glc6P	-2.043	-5.490 to 1.403	No	ns	0.3333
control vs. 100 µM Glc6P	-4.723	-8.170 to -1.277	Yes	**	0.0060
sigRep					
control vs. 20 µM Glc6P	-15.87	-19.32 to -12.43	Yes	****	<0.0001
control vs. 50 µM Glc6P	-14.45	-17.89 to -11.00	Yes	****	<0.0001
control vs. 100 µM Glc6P	-5.840	-9.286 to -2.394	Yes	***	0.0008
gunSOS1					
control vs. 20 µM Glc6P	-0.01000	-3.456 to 3.436	No	ns	>0.9999
control vs. 50 µM Glc6P	-0.1200	-3.566 to 3.326	No	ns	0.9995
control vs. 100 µM Glc6P	-0.4400	-3.886 to 3.006	No	ns	0.9777

**Test details**	**Mean 1**	**Mean 2**	**Mean Diff.**	**SE of diff.**	**N1**	**N2**	**q**	**DF**
WT								
control vs. 20 µM Glc6P	1.000	0.9667	0.03333	1.375	3	3	0.02425	24.00
control vs. 50 µM Glc6P	1.000	3.043	-2.043	1.375	3	3	1.486	24.00
control vs. 100 µM Glc6P	1.000	5.723	-4.723	1.375	3	3	3.436	24.00
sigRep								
control vs. 20 µM Glc6P	14.24	30.11	-15.87	1.375	3	3	11.55	24.00
control vs. 50 µM Glc6P	14.24	28.68	-14.45	1.375	3	3	10.51	24.00
control vs. 100 µM Glc6P	14.24	20.08	-5.840	1.375	3	3	4.248	24.00
gunSOS1								
control vs. 20 µM Glc6P	0.3100	0.3200	-0.01000	1.375	3	3	0.007274	24.00
control vs. 50 µM Glc6P	0.3100	0.4300	-0.1200	1.375	3	3	0.08729	24.00
control vs. 100 µM Glc6P	0.3100	0.7500	-0.4400	1.375	3	3	0.3201	24.00

Supplementary Table 13. Statistical analyses for $GPX5_{cp}$ expression following treatment with glucose 6-phosphate (Glc6P), presented in Fig. 6e. *P*, probability (significance test); *SS*, sum of squares; *DF*, degree of freedom; *MS*, means square; *F*, F-value (Fisher test); *DFn*, degrees of freedom in the numerator; *DFd*, degrees of freedom in the denominator; *SE*, standard error of the sample mean; *N*, sample number.

**Two-way ANOVA Ordinary**					
Alpha	0.05				
**Source of Variation**	**% of total variation**	**P value**	**P value summary**	**Significant?**	
Interaction	7.804	<0.0001	****	Yes	
strain	87.70	<0.0001	****	Yes	
metabolite	3.787	<0.0001	****	Yes	
**ANOVA table**	**SS**	**DF**	**MS**	**F (DFn, DFd)**	**P value**
Interaction	152.6	6	25.44	F (6, 24) = 43.97	P<0.0001
strain	1715	2	857.6	F (2, 24) = 1482	P<0.0001
metabolite	74.06	3	24.69	F (3, 24) = 42.67	P<0.0001
Residual	13.88	24	0.5785		

**Data summary**	
Number of columns (metabolite)	4
Number of rows (strain)	3
Number of values	36
**Two-way ANOVA Multiple comparison**	
Number of families	3
Number of comparisons per family	3
Alpha	0.05

**Dunnett's multiple comparisons test**	**Mean Diff.**	**95.00% CI of diff.**	**Below threshold?**	**Summary**	**Adjusted P Value**
WT					
control vs. 20 µM Glc6P	-0.1600	-1.717 to 1.397	No	ns	0.9880
control vs. 50 µM Glc6P	-2.200	-3.757 to -0.6432	Yes	**	0.0046
control vs. 100 µM Glc6P	-6.083	-7.640 to -4.527	Yes	****	<0.0001
sigRep					
control vs. 20 µM Glc6P	-9.450	-11.01 to -7.893	Yes	****	<0.0001
control vs. 50 µM Glc6P	-7.100	-8.657 to -5.543	Yes	****	<0.0001
control vs. 100 µM Glc6P	-3.617	-5.173 to -2.060	Yes	****	<0.0001
gunSOS1					
control vs. 20 µM Glc6P	-0.04000	-1.597 to 1.517	No	ns	0.9999
control vs. 50 µM Glc6P	-0.2700	-1.827 to 1.287	No	ns	0.9478
control vs. 100 µM Glc6P	-0.7867	-2.343 to 0.7701	No	ns	0.4570

**Test details**	**Mean 1**	**Mean 2**	**Mean Diff.**	**SE of diff.**	**N1**	**N2**	**q**	**DF**
WT								
control vs. 20 µM Glc6P	1.000	1.160	-0.1600	0.6210	3	3	0.2576	24.00
control vs. 50 µM Glc6P	1.000	3.200	-2.200	0.6210	3	3	3.543	24.00
control vs. 100 µM Glc6P	1.000	7.083	-6.083	0.6210	3	3	9.796	24.00
sigRep								
control vs. 20 µM Glc6P	11.34	20.79	-9.450	0.6210	3	3	15.22	24.00
control vs. 50 µM Glc6P	11.34	18.44	-7.100	0.6210	3	3	11.43	24.00
control vs. 100 µM Glc6P	11.34	14.96	-3.617	0.6210	3	3	5.824	24.00
gunSOS1								
control vs. 20 µM Glc6P	0.4000	0.4400	-0.04000	0.6210	3	3	0.06441	24.00
control vs. 50 µM Glc6P	0.4000	0.6700	-0.2700	0.6210	3	3	0.4348	24.00
control vs. 100 µM Glc6P	0.4000	1.187	-0.7867	0.6210	3	3	1.267	24.00

Supplementary Table 14. Statistical analyses for *$GPX5_{cyt}$* expression following treatment with aconitate, presented in Fig. 6f. *P*, probability (significance test); *SS*, sum of squares; *DF*, degree of freedom; *MS*, means square; *F*, F-value (Fisher test); *DFn*, degrees of freedom in the numerator; *DFd*, degrees of freedom in the denominator; *SE*, standard error of the sample mean; *N*, sample number.

**Two-way ANOVA Ordinary**					
Alpha	0.05				
**Source of Variation**	**% of total variation**	**P value**	**P value summary**	**Significant?**	
Interaction	22.90	<0.0001	****	Yes	
strain	31.91	<0.0001	****	Yes	
metabolite	42.02	<0.0001	****	Yes	
**ANOVA table**	**SS**	**DF**	**MS**	**F (DFn, DFd)**	**P value**
Interaction	158.3	6	26.38	F (6, 24) = 28.93	P<0.0001
strain	220.6	2	110.3	F (2, 24) = 120.9	P<0.0001
metabolite	290.5	3	96.83	F (3, 24) = 106.2	P<0.0001
Residual	21.88	24	0.9119		

**Data summary**	
Number of columns (metabolite)	4
Number of rows (strain)	3
Number of values	36

**Two-way ANOVA Multiple comparison**	
Number of families	3
Number of comparisons per family	3
Alpha	0.05

**Dunnett's multiple comparisons test**	**Mean Diff.**	**95.00% CI of diff.**	**Below threshold?**	**Summary**	**Adjusted P Value**
WT					
control vs. 20 µM Acon	-0.1767	-2.131 to 1.778	No	ns	0.9918
control vs. 50 µM Acon	-0.6067	-2.561 to 1.348	No	ns	0.7781
control vs. 100 µM Acon	0.1033	-1.851 to 2.058	No	ns	0.9983
sigRep					
control vs. 20 µM Acon	-8.807	-10.76 to -6.852	Yes	****	<0.0001
control vs. 50 µM Acon	0.6467	-1.308 to 2.601	No	ns	0.7453
control vs. 100 µM Acon	3.903	1.949 to 5.858	Yes	***	0.0001
gunSOS1					
control vs. 20 µM Acon	-9.167	-11.12 to -7.212	Yes	****	<0.0001
control vs. 50 µM Acon	-0.3567	-2.311 to 1.598	No	ns	0.9401
control vs. 100 µM Acon	-0.1867	-2.141 to 1.768	No	ns	0.9903

**Test details**	**Mean 1**	**Mean 2**	**Mean Diff.**	**SE of diff.**	**N1**	**N2**	**q**	**DF**
WT								
control vs. 20 µM Acon	1.000	1.177	-0.1767	0.7797	3	3	0.2266	24.00
control vs. 50 µM Acon	1.000	1.607	-0.6067	0.7797	3	3	0.7781	24.00
control vs. 100 µM Acon	1.000	0.8967	0.1033	0.7797	3	3	0.1325	24.00
sigRep								
control vs. 20 µM Acon	6.030	14.84	-8.807	0.7797	3	3	11.30	24.00
control vs. 50 µM Acon	6.030	5.383	0.6467	0.7797	3	3	0.8294	24.00
control vs. 100 µM Acon	6.030	2.127	3.903	0.7797	3	3	5.006	24.00
gunSOS1								
control vs. 20 µM Acon	0.5867	9.753	-9.167	0.7797	3	3	11.76	24.00
control vs. 50 µM Acon	0.5867	0.9433	-0.3567	0.7797	3	3	0.4574	24.00
control vs. 100 µM Acon	0.5867	0.7733	-0.1867	0.7797	3	3	0.2394	24.00

Supplementary Table 15. Statistical analyses for $\mathit{GPX5}_{cp}$ expression following treatment with aconitate, presented in Fig. 6f. *P*, probability (significance test); *SS*, sum of squares; *DF*, degree of freedom; *MS*, means square; *F*, F-value (Fisher test); *DFn*, degrees of freedom in the numerator; *DFd*, degrees of freedom in the denominator; *SE*, standard error of the sample mean; *N*, sample number.

**Two-way ANOVA Ordinary**					
Alpha	0.05				
**Source of Variation**	**% of total variation**	**P value**	**P value summary**	**Significant?**	
Interaction	30.75	<0.0001	****	Yes	
strain	27.59	<0.0001	****	Yes	
metabolite	39.86	<0.0001	****	Yes	
**ANOVA table**	SS	DF	MS	F (DFn, DFd)	P value
Interaction	31.93	6	5.321	F (6, 24) = 68.18	P<0.0001
strain	28.65	2	14.32	F (2, 24) = 183.5	P<0.0001
metabolite	41.40	3	13.80	F (3, 24) = 176.8	P<0.0001
Residual	1.873	24	0.07804		
**Data summary**					
Number of columns (metabolite)	4				
Number of rows (strain)	3				
Number of values	36				
**Two-way ANOVA Multiple comparison**					
Number of families	3				
Number of comparisons per family	3				
Alpha	0.05				

**Dunnett's multiple comparisons test**	**Mean Diff.**	**95.00% CI of diff.**	**Below threshold?**	**Summary**	**Adjusted P Value**
WT					
control vs. 20 µM Acon	-0.1233	-0.6951 to 0.4485	No	ns	0.9072
control vs. 50 µM Acon	-0.6267	-1.198 to -0.05485	Yes	*	0.0296
control vs. 100 µM Acon	0.1667	-0.4051 to 0.7385	No	ns	0.8072
sigRep					
control vs. 20 µM Acon	-3.330	-3.902 to -2.758	Yes	****	<0.0001
control vs. 50 µM Acon	1.510	0.9382 to 2.082	Yes	****	<0.0001
control vs. 100 µM Acon	2.450	1.878 to 3.022	Yes	****	<0.0001
gunSOS1					
control vs. 20 µM Acon	-2.660	-3.232 to -2.088	Yes	****	<0.0001
control vs. 50 µM Acon	-0.1900	-0.7618 to 0.3818	No	ns	0.7430
control vs. 100 µM Acon	-0.2067	-0.7785 to 0.3651	No	ns	0.6948

**Test details**	**Mean 1**	**Mean 2**	**Mean Diff.**	**SE of diff.**	**N1**	**N2**	**q**	**DF**
WT								
control vs. 20 µM Acon	1.000	1.123	-0.1233	0.2281	3	3	0.5407	24.00
control vs. 50 µM Acon	1.000	1.627	-0.6267	0.2281	3	3	2.747	24.00
control vs. 100 µM Acon	1.000	0.8333	0.1667	0.2281	3	3	0.7307	24.00
sigRep								
control vs. 20 µM Acon	3.130	6.460	-3.330	0.2281	3	3	14.60	24.00
control vs. 50 µM Acon	3.130	1.620	1.510	0.2281	3	3	6.620	24.00
control vs. 100 µM Acon	3.130	0.6800	2.450	0.2281	3	3	10.74	24.00
gunSOS1								
control vs. 20 µM Acon	0.2567	2.917	-2.660	0.2281	3	3	11.66	24.00
control vs. 50 µM Acon	0.2567	0.4467	-0.1900	0.2281	3	3	0.8330	24.00
control vs. 100 µM Acon	0.2567	0.4633	-0.2067	0.2281	3	3	0.9060	24.00

Supplementary Table 16. Statistical analyses for *SAK1* expression following treatment with aconitate, presented in Fig. 6g. *P*, probability (significance test); *SS*, sum of squares; *DF*, degree of freedom; *MS*, means square; *F*, F-value (Fisher test); *DFn*, degrees of freedom in the numerator; *DFd*, degrees of freedom in the denominator; *SE*, standard error of the sample mean; *N*, sample number.

**Two-way ANOVA Ordinary**					
Alpha	0.05				
**Source of Variation**	**% of total variation**	**P value**	**P value summary**	**Significant?**	
Interaction	49.81	<0.0001	****	Yes	
strain	12.86	<0.0001	****	Yes	
metabolite	35.02	<0.0001	****	Yes	
**ANOVA table**	**SS**	**DF**	**MS**	**F (DFn, DFd)**	**P value**
Interaction	15402	6	2567	F (6, 24) = 86.01	P<0.0001
strain	3976	2	1988	F (2, 24) = 66.62	P<0.0001
metabolite	10830	3	3610	F (3, 24) = 121.0	P<0.0001
Residual	716.3	24	29.84		

**Data summary**	
Number of columns (metabolite)	4
Number of rows (strain)	3
Number of values	36
**Two-way ANOVA Multiple comparison**	
Number of families	3
Number of comparisons per family	3
Alpha	0.05

**Dunnett's multiple comparisons test**	**Mean Diff.**	**95.00% CI of diff.**	**Below threshold?**	**Summary**	**Adjusted P Value**
WT					
control vs. 20 µM Acon	-0.2367	-11.42 to 10.95	No	ns	>0.9999
control vs. 50 µM Acon	-3.273	-14.46 to 7.909	No	ns	0.8052
control vs. 100 µM Acon	-2.583	-13.77 to 8.599	No	ns	0.8895
sigRep					
control vs. 20 µM Acon	-16.06	-27.25 to -4.881	Yes	**	0.0040
control vs. 50 µM Acon	-5.853	-17.04 to 5.329	No	ns	0.4296
control vs. 100 µM Acon	2.253	-8.929 to 13.44	No	ns	0.9222
gunSOS1					
control vs. 20 µM Acon	-107.2	-118.4 to -96.01	Yes	****	<0.0001
control vs. 50 µM Acon	-0.9400	-12.12 to 10.24	No	ns	0.9933
control vs. 100 µM Acon	-0.6300	-11.81 to 10.55	No	ns	0.9979

**Test details**	**Mean 1**	**Mean 2**	**Mean Diff.**	**SE of diff.**	**N1**	**N2**	**q**	**DF**
WT								
control vs. 20 µM Acon	1.000	1.237	-0.2367	4.461	3	3	0.05306	24.00
control vs. 50 µM Acon	1.000	4.273	-3.273	4.461	3	3	0.7338	24.00
control vs. 100 µM Acon	1.000	3.583	-2.583	4.461	3	3	0.5792	24.00
sigRep								
control vs. 20 µM Acon	7.743	23.81	-16.06	4.461	3	3	3.601	24.00
control vs. 50 µM Acon	7.743	13.60	-5.853	4.461	3	3	1.312	24.00
control vs. 100 µM Acon	7.743	5.490	2.253	4.461	3	3	0.5052	24.00
gunSOS1								
control vs. 20 µM Acon	0.8933	108.1	-107.2	4.461	3	3	24.03	24.00
control vs. 50 µM Acon	0.8933	1.833	-0.9400	4.461	3	3	0.2107	24.00
control vs. 100 µM Acon	0.8933	1.523	-0.6300	4.461	3	3	0.1412	24.00

Supplementary Table 17. Statistical analyses for *PSBP2* expression following treatment with aconitate, presented in Fig. 6g. *P*, probability (significance test); *SS*, sum of squares; *DF*, degree of freedom; *MS*, means square; *F*, F-value (Fisher test); *DFn*, degrees of freedom in the numerator; *DFd*, degrees of freedom in the denominator; *SE*, standard error of the sample mean; *N*, sample number.

**Two-way ANOVA Ordinary**					
Alpha	0.05				
**Source of Variation**	**% of total variation**	**P value**	**P value summary**	**Significant?**	
Interaction	25.51	<0.0001	****	Yes	
strain	47.78	<0.0001	****	Yes	
metabolite	25.30	<0.0001	****	Yes	
**ANOVA table**	**SS**	**DF**	**MS**	**F (DFn, DFd)**	**P value**
Interaction	161.1	6	26.85	F (6, 24) = 72.08	P<0.0001
strain	301.7	2	150.9	F (2, 24) = 405.0	P<0.0001
metabolite	159.8	3	53.26	F (3, 24) = 143.0	P<0.0001
Residual	8.940	24	0.3725		

**Data summary**	
Number of columns (metabolite)	4
Number of rows (strain)	3
Number of values	36

**Two-way ANOVA Multiple comparison**	
Number of families	3
Number of comparisons per family	3
Alpha	0.05

**Dunnett's multiple comparisons test**	**Mean Diff.**	**95.00% CI of diff.**	**Below threshold?**	**Summary**	**Adjusted P Value**
WT					
control vs. 20 µM Acon	-0.3700	-1.619 to 0.8792	No	ns	
control vs. 50 µM Acon	-1.770	-3.019 to -0.5208	Yes	**	
control vs. 100 µM Acon	-1.697	-2.946 to -0.4474	Yes	**	
sigRep					
control vs. 20 µM Acon	-7.653	-8.903 to -6.404	Yes	****	
control vs. 50 µM Acon	0.3600	-0.8892 to 1.609	No	ns	
control vs. 100 µM Acon	5.030	3.781 to 6.279	Yes	****	
gunSOS1					
control vs. 20 µM Acon	-5.497	-6.746 to -4.247	Yes	****	
control vs. 50 µM Acon	-0.1767	-1.426 to 1.073	No	ns	
control vs. 100 µM Acon	-0.1600	-1.409 to 1.089	No	ns	

**Test details**	**Mean 1**	**Mean 2**	**Mean Diff.**	**SE of diff.**	**N1**	**N2**	**q**	**DF**
WT								
control vs. 20 µM Acon	1.000	1.370	-0.3700	0.4983	3	3	0.7425	24.00
control vs. 50 µM Acon	1.000	2.770	-1.770	0.4983	3	3	3.552	24.00
control vs. 100 µM Acon	1.000	2.697	-1.697	0.4983	3	3	3.405	24.00
sigRep								
control vs. 20 µM Acon	7.333	14.99	-7.653	0.4983	3	3	15.36	24.00
control vs. 50 µM Acon	7.333	6.973	0.3600	0.4983	3	3	0.7224	24.00
control vs. 100 µM Acon	7.333	2.303	5.030	0.4983	3	3	10.09	24.00
gunSOS1								
control vs. 20 µM Acon	0.1167	5.613	-5.497	0.4983	3	3	11.03	24.00
control vs. 50 µM Acon	0.1167	0.2933	-0.1767	0.4983	3	3	0.3545	24.00
control vs. 100 µM Acon	0.1167	0.2767	-0.1600	0.4983	3	3	0.3211	24.00

Supplementary Table 18. Statistical analyses for *MBS* expression following treatment with aconitate, presented in Fig. 6g. *P*, probability (significance test); *SS*, sum of squares; *DF*, degree of freedom; *MS*, means square; *F*, F-value (Fisher test); *DFn*, degrees of freedom in the numerator; *DFd*, degrees of freedom in the denominator; *SE*, standard error of the sample mean; *N*, sample number.

**Two-way ANOVA Ordinary**					
Alpha	0.05				
**Source of Variation**	**% of total variation**	**P value**	**P value summary**	**Significant?**	
Interaction	28.05	<0.0001	****	Yes	
strain	32.20	<0.0001	****	Yes	
metabolite	37.62	<0.0001	****	Yes	
**ANOVA table**	**SS**	**DF**	**MS**	**F (DFn, DFd)**	**P value**
Interaction	1424	6	237.3	F (6, 24) = 52.66	P<0.0001
strain	1635	2	817.4	F (2, 24) = 181.4	P<0.0001
metabolite	1910	3	636.5	F (3, 24) = 141.2	P<0.0001
Residual	108.2	24	4.507		

**Data summary**	
Number of columns (metabolite)	4
Number of rows (strain)	3
Number of values	36
**Two-way ANOVA Multiple comparison**	
Number of families	3
Number of comparisons per family	3
Alpha	0.05

**Dunnett's multiple comparisons test**	**Mean Diff.**	**95.00% CI of diff.**	**Below threshold?**	**Summary**	**Adjusted P Value**
WT					
control vs. 20 µM Acon	-0.4133	-4.759 to 3.932	No	ns	0.9904
control vs. 50 µM Acon	-4.537	-8.882 to -0.1912	Yes	*	0.0394
control vs. 100 µM Acon	-3.460	-7.806 to 0.8855	No	ns	0.1395
sigRep					
control vs. 20 µM Acon	-25.88	-30.23 to -21.54	Yes	****	<0.0001
control vs. 50 µM Acon	-14.28	-18.63 to -9.934	Yes	****	<0.0001
control vs. 100 µM Acon	1.287	-3.059 to 5.632	No	ns	0.8002
gunSOS1					
control vs. 20 µM Acon	-28.43	-32.78 to -24.08	Yes	****	<0.0001
control vs. 50 µM Acon	-0.2200	-4.566 to 4.126	No	ns	0.9985
control vs. 100 µM Acon	-0.3267	-4.672 to 4.019	No	ns	0.9952

**Test details**	**Mean 1**	**Mean 2**	**Mean Diff.**	**SE of diff.**	**N1**	**N2**	**q**	**DF**
WT								
control vs. 20 µM Acon	1.000	1.413	-0.4133	1.733	3	3	0.2384	24.00
control vs. 50 µM Acon	1.000	5.537	-4.537	1.733	3	3	2.617	24.00
control vs. 100 µM Acon	1.000	4.460	-3.460	1.733	3	3	1.996	24.00
sigRep								
control vs. 20 µM Acon	9.413	35.30	-25.88	1.733	3	3	14.93	24.00
control vs. 50 µM Acon	9.413	23.69	-14.28	1.733	3	3	8.238	24.00
control vs. 100 µM Acon	9.413	8.127	1.287	1.733	3	3	0.7423	24.00
gunSOS1								
control vs. 20 µM Acon	0.4633	28.89	-28.43	1.733	3	3	16.40	24.00
control vs. 50 µM Acon	0.4633	0.6833	-0.2200	1.733	3	3	0.1269	24.00
control vs. 100 µM Acon	0.4633	0.7900	-0.3267	1.733	3	3	0.1884	24.00

Supplementary Table 19. Statistical analyses for *ACH1* expression following treatment with aconitate, presented in Fig. 6h. *P*, probability (significance test); *SS*, sum of squares; *DF*, degree of freedom; *MS*, means square; *F*, F-value (Fisher test); *DFn*, degrees of freedom in the numerator; *DFd*, degrees of freedom in the denominator; *SE*, standard error of the sample mean; *N*, sample number; *t ratio*, the difference between sample means divided by the standard error of the difference; *df*, degrees of freedom.

Column A	control
vs.	vs.
Column B	20 µM Acon
Test details	
Test name	Unpaired t test
Variance assumption	Individual variance for each row
Multiple comparisons	False Discovery Rate (FDR)
Method	Two-stage step-up (Benjamini, Krieger, and Yekutieli)
Desired FDR (Q)	1.00%
Number of tests performed	3
Number of rows omitted	0

	Mean of control	Mean of 20 µM Acon	Difference	SE of difference	t ratio	df	*P* value
WT	1.000	0.9967	0.003333	0.08192	0.04069	4.000	0.979188
sigRep	0.1767	0.1600	0.01667	0.02261	0.7372	4.000	0.760378
gunSOS1	0.06333	0.1500	-0.08667	0.01563	5.543	4.000	0.015694

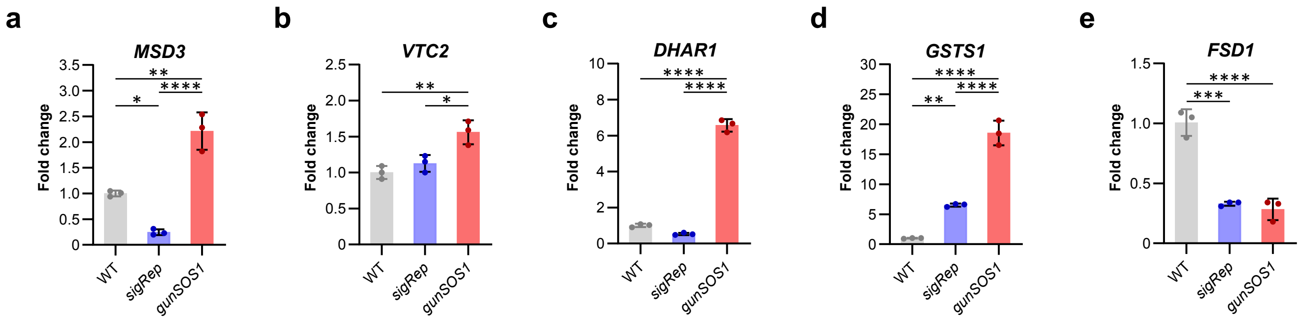

Supplementary Fig. 11 Expression of genes, which induction was previously associated with H_2O_2 or organic peroxides, but not with $^1O_2$. a *Mn SUPEROXIDE DISMUTASE 3* (*MSD3*). **b** GDP-L-galactose *PHOSPHORYLASE* (*VTC2*). **c** *DEHYDROASCORBATE REDUCTASE* (*DHAR1*). **d** *GLUTATHIONE S-TRANSFERASE* (*GSTS1*). **e** Fe *SUPEROXIDE DISMUTASE* (*FSD1*). Samples were collected after 2 h following transfer from dark to light. Experiments were performed in biological replications ($n = 3$); results are presented as as a fold change ($2^{-\Delta\Delta Ct}$) normalized to the mean of Ct_{exp}-Ct_{ref} of WT; the error bars represent calculated ±SD. Significant differences were calculated using one-way ANOVA, pair-wise comparison with the Tukey's post-hoc test (non-significant not shown), $^*P < 0.05$, $^{**}P < 0.01$, $^{***}P < 0.001$, and $^{****}P < 0.0001$.

Supplementary Table 20. Primers used in this study.

**v5.5 gene ID**	**Gene name**	**Forward (5’ → 3’)**	**Reverse (5’ → 3’)**
***GPX5-ARS2* fusion**			
Cre10.g458450	*GPX5 5’RR*	CTCGAGGGTACATGTTTAGAACCCGCT*	GATATCTGCAATCGTCGCTGGTTC*
**Rescue with genomic *TSPP1* (BAC PTQ5987 template)**			
Cre12.g497750	*TSPP1*	GATGAAGCTCAGGGCGAGAC	CAGCAGCAGTAGCGAACCAA
**qRT-PCR**			
Reference gene for all qRT-PCR	*18S rRNA*	GATGGCTACCACATCCAAGGAA[1]	AAGCGCCCGGTATTGTTATTTATT[1]
Cre10.g458450	*GPX5*$_{cyt}$	GCGGTCGCCAATAACCAAT[1]	AAGGGCTGTCCCGAAAGC[1]
Cre10.g458450	*GPX5*$_{cp}$	AACCCTTTCACTCACATGCTGTCT[1]	CGAGCGGCGACAGGAGTA[1]
	GPX5-ARS2	GCCTTTGCATCTACTGAACCAG	TCCCAAACGATCCCTTGACAG
Cre12.g497750	*TSPP1*	GAAGATAGCAATGACGGCAAGG	ATGCTTCGTCCTTCTCTGACTG
Cre12.g497750	*TSPP1* (kinetics)	AGGACGAAGCATACCTCAAGTG	TGAGCGTGCCATCATAGTCTAG
Cre16.g678851	*PSBP2*	AAGCTGTACGAGTACGAGTACG	CTTGTATGCCGCGTTTAGGATG
Cre09.g416500	*MBS*	TGCGCGGATCTACCAAGAAG	CGGAAGCGAAGTGTACATCTTC
Cre17.g741300	*SAK1*	TCAAGCGTGTGGGTAAGAGCTA[2]	ACGCTATCTCCGTCCTAATCCA[2]
Cre06.g281250	*CFA1*	CCTACAACGACAACGACGTG[2]	GGAAGTTCCAGGATGACCAG[2]
Cre09.g398700	*CFA2* (*CPLD27*)	GTCCATTGAGATGTTCGAGCAC	ATGTGCACGAACAACTTGCC
Cre06.g299700	*SOUL1*	TGAAGAAGATCCCCATGACTGC	CGAAGAACGACACCTTGAAGTG
Cre08.g380300	*MSRA3*	GACTGAAGTTGGCGACGTTC	AGTCGTAGTTGGGGTTCTTGTC
Cre14.g623650	*ADH7*	AAGAAGGTCGTTGGCTCCATC	TAGCCTCGTTCACCTTGCTG
Cre16.g683400	*RABPR1*	AGTGGGCCATCAAATGCATG	TGATGTCCAGGAACAGCTTGG
Cre06.g263550	*LCI7*	TTTGGTTGCGTTGCATGTAT[2]	TCAACGCGGTGTCAAACTTA[2]
Cre16.g676150	*MSD3*	TCGACAACGAGACCATGTTCC	ACAATCTCCGACAGCGACAG
Cre13.g588150	*VTC2*	CAACCAGCCGTTCAACATCATC	CCGCAATCTCAAACGATGCC
Cre10.g456750	*DHAR1*	GACAGCGATGTCATTGTGGTG	AACAGTTTGGCGCCGATTTC
Cre16.g688550	*GSTS1*	TGTTGTTCCACATCGGCAAC	CCTGGCCAAATGGGAACTTTG
Cre10.g436050	*FSD1*	TTCTTCTGGGAGAGCATGAAGC	ACTCCTCCTTGAACTTGTCCAG
Cre01.g020223	*FUM2*	GCAGATGAGTTTGCAGGCATC	TACTTCACCTGCGCTGCATAG
Cre17.g732802		ATCCAGAAGTGGGAGTACGTG	ATCCAACGTCACCACCCATG
Cre01.g042750	*ACH1*	CCATGTTCCCTTACAACAAGCG	TTCAGGTGCTCCTTGAACGAG

*Underlined sequences indicate restriction sites, XhoI (CTCGAG) and EcoRV (GATATC)

Supplementary Table 21. Initial transcript analysis of *GPX5* (Fig. 1b) and *GPX5-ARS2* (Fig. 1c) indicated ≤ 0.9 change in quantification cycle (Cq) between sigRep and WT, both in dark and subsequent exposure to light for 2 h. t [min] 0 indicates dark condition, t [min] 120 indicates subsequent exposure to light for 2 h; Ct_A-C, biological replications (*n* = 3); Ct_{AV_WT}, calculated average Ct in WT in a given conditions; Ct_L-Ct_0, Ct change upon shift from dark to light; Ct_{AV_WT}-Ct_{AV_sigRep}, calculated difference between averaged Ct in WT and *sigRep* in the same condition.

<table>
<tr><td colspan="12">GPX5</td></tr>
<tr><td></td><td colspan="5">WT</td><td colspan="5">sigRep</td><td rowspan="2">Ct_{AV_WT}-Ct_{AV_sigRep}</td></tr>
<tr><td>t [min]</td><td>Ct_A</td><td>Ct_B</td><td>Ct_C</td><td>Ct_{AV_WT}</td><td>Ct_L-Ct_0</td><td>Ct_A</td><td>Ct_B</td><td>Ct_C</td><td>Ct_{AV_sigRep}</td><td>Ct_L-Ct_0</td></tr>
<tr><td>0</td><td>13.13</td><td>13.19</td><td>13.55</td><td>13.29</td><td rowspan="2">3</td><td>13.03</td><td>12.93</td><td>12.6</td><td>12.85</td><td rowspan="2">2.57</td><td>0.44</td></tr>
<tr><td>120</td><td>16.31</td><td>16.23</td><td>16.34</td><td>16.29</td><td>15.35</td><td>15.52</td><td>15.39</td><td>15.42</td><td>0.87</td></tr>
<tr><td colspan="12">GPX5-ARS2</td></tr>
<tr><td></td><td colspan="5">WT</td><td colspan="5">sigRep</td><td rowspan="2">Ct_{AV_WT}-Ct_{AV_sigRep}</td></tr>
<tr><td>t [min]</td><td>Ct_A</td><td>Ct_B</td><td>Ct_C</td><td>Ct_{AV_WT}</td><td>Ct_L-Ct_0</td><td>Ct_A</td><td>Ct_B</td><td>Ct_C</td><td>Ct_{AV_sigRep}</td><td>Ct_L-Ct_0</td></tr>
<tr><td>0</td><td>14.53</td><td>15.04</td><td>15.03</td><td>14.87</td><td rowspan="2">2.17</td><td>13.54</td><td>13.88</td><td>13.74</td><td>14.18</td><td rowspan="2">3.28</td><td>0.69</td></tr>
<tr><td>120</td><td>16.81</td><td>17.09</td><td>17.21</td><td>17.04</td><td>15.5</td><td>17.31</td><td>17.49</td><td>17.46</td><td>-0.42</td></tr>
</table>

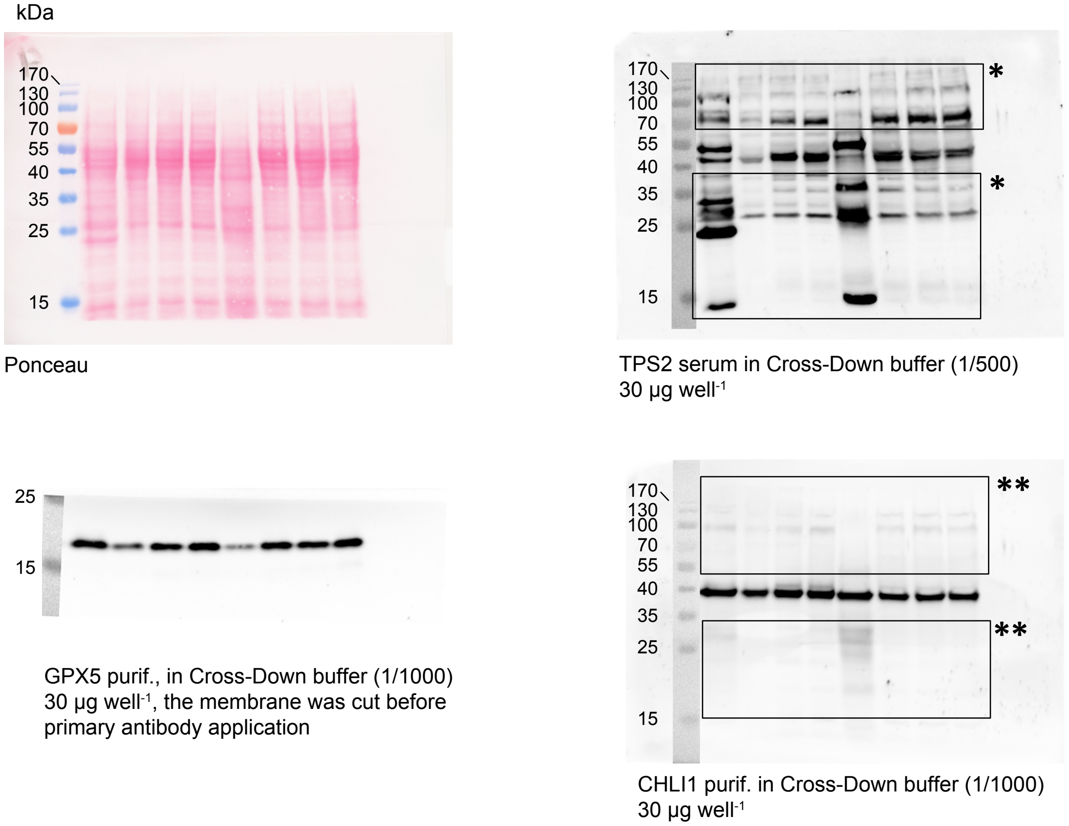

Supplementary Fig. 12 Supplementary material for western blots in Figure 2d. Proteins were extracted as described in Methods, followed by separation on 12% SDS-PAGE and transfer to the nitrocellulose membrane. Unspecific immunoreactions are indicated by an asterisk; incomplete stripping after the previous immunoreaction are indicated by a double asterisk. In all cases PageRuler Prestained Protein Ladder #26616 (Thermo Fisher Scientific, Waltham, MA, USA) was used as a reference.

Supplementary References

1 Fischer, B. B. *et al.* Function and regulation of the glutathione peroxidase homologous gene GPXH/GPX5 in Chlamydomonas reinhardtii. *Plant Mol. Biol.* **71**, 569-583, doi:10.1007/s11103-009-9540-8 (2009).

2 Wakao, S. *et al.* Phosphoprotein SAK1 is a regulator of acclimation to singlet oxygen in Chlamydomonas reinhardtii. *eLife* **3**, e02286, doi:10.7554/eLife.02286 (2014).
