# Supplementary material for: Singlet oxygen-induced signalling depends on the metabolic status of the Chlamydomonas reinhardtii cell
Source: Commun Biol. 2023 May 16;6:529. doi: 10.1038/s42003-023-04872-5 (PMC10188600; doi:10.1038/s42003-023-04872-5)
Supplement: Supplementary file 2 — Description of Additional Supplementary Files [file 42003_2023_4872_MOESM2_ESM.pdf]

Description of Additional Supplementary Files

File name: Supplementary Data 1

Description: Co-expression of genes in gunSOS1 and sigRep, based on the RNA-seq results.

File name: Supplementary Data 2

Description: Differentially expressed genes in gunSOS1 compared to sigRep, based on the RNA-seq

File name: Supplementary Data 3

Description: KEGG enrichment of genes showing differential expression in gunSOS1 compared to sigRep

File name: Supplementary Data 4

Description: The numerical source data behind the graphs presented in the main text
