# Supplementary material for: Singlet oxygen-induced signalling depends on the metabolic status of the Chlamydomonas reinhardtii cell
Source: Commun Biol. 2023 May 16;6:529. doi: 10.1038/s42003-023-04872-5 (PMC10188600; doi:10.1038/s42003-023-04872-5)
Supplement: Supplementary file 7 — Reporting Summary [file 42003_2023_4872_MOESM7_ESM.pdf]

nature portfolio

Corresponding author(s): Pawel Brzezowski

Last updated by author(s): Apr 13, 2023

Reporting Summary

Nature Portfolio wishes to improve the reproducibility of the work that we publish. This form provides structure for consistency and transparency in reporting. For further information on Nature Portfolio policies, see our Editorial Policies and the Editorial Policy Checklist.

Statistics

For all statistical analyses, confirm that the following items are present in the figure legend, table legend, main text, or Methods section.

n/a	Confirmed	
☐	☒	The exact sample size (*n*) for each experimental group/condition, given as a discrete number and unit of measurement
☐	☒	A statement on whether measurements were taken from distinct samples or whether the same sample was measured repeatedly
☐	☒	The statistical test(s) used AND whether they are one- or two-sided<br>*Only common tests should be described solely by name; describe more complex techniques in the Methods section.*
☒	☐	A description of all covariates tested
☒	☐	A description of any assumptions or corrections, such as tests of normality and adjustment for multiple comparisons
☐	☒	A full description of the statistical parameters including central tendency (e.g. means) or other basic estimates (e.g. regression coefficient) AND variation (e.g. standard deviation) or associated estimates of uncertainty (e.g. confidence intervals)
☐	☒	For null hypothesis testing, the test statistic (e.g. *F*, *t*, *r*) with confidence intervals, effect sizes, degrees of freedom and *P* value noted<br>*Give P values as exact values whenever suitable.*
☒	☐	For Bayesian analysis, information on the choice of priors and Markov chain Monte Carlo settings
☒	☐	For hierarchical and complex designs, identification of the appropriate level for tests and full reporting of outcomes
☒	☐	Estimates of effect sizes (e.g. Cohen's *d*, Pearson's *r*), indicating how they were calculated

Our web collection on statistics for biologists contains articles on many of the points above.

Software and code

Policy information about availability of computer code

Data collection	DNA and RNA sequence data were collected from JGI (DOE Joint Genome Institute,Walnut Creek, CA, USA, https://phytozome-next.jgi.doe.gov/); chemiluminescence signal was detected using Chemiluminescence Imager (ECL, Chemostar Series-ChemoCam (Intas Science Imaging Instruments GmbH, Göttingen, Germany); HPLC data were collected using ChemStation software (Agilent Technologies GmbH, Germany).
Data analysis	DNA and RNA sequence data were analyzed using Vector NTI (Invitrogen); chemiluminescence signal was analyzed using ChemoStarTS software Intas Science Imaging Instruments GmbH, Göttingen, Germany, HPLC data was analyzed using ChemStation software (Agilent Technologies GmbH, Germany) and Microsoft Excel 2010; Statistical analyses were performed using GraphPad Prism (GraphPad Software, LLC San Diego, CA, USA).

For manuscripts utilizing custom algorithms or software that are central to the research but not yet described in published literature, software must be made available to editors and reviewers. We strongly encourage code deposition in a community repository (e.g. GitHub). See the Nature Portfolio guidelines for submitting code & software for further information.

Data

Policy information about availability of data

All manuscripts must include a data availability statement. This statement should provide the following information, where applicable:

- Accession codes, unique identifiers, or web links for publicly available datasets
- A description of any restrictions on data availability
- For clinical datasets or third party data, please ensure that the statement adheres to our policy

The authors declare no restrictions on data availability and all relevant data supporting the findings of this study are included in the manuscript or its supplementary material. The raw data can be made available upon reasonable request from the corresponding author. The DNA/RNA sequence is publicly available at https://phytozome-next.jgi.doe.gov/

Human research participants

Policy information about studies involving human research participants and Sex and Gender in Research.

Reporting on sex and gender	not applicable
Population characteristics	not applicable
Recruitment	not applicable
Ethics oversight	not applicable

Note that full information on the approval of the study protocol must also be provided in the manuscript.

Field-specific reporting

Please select the one below that is the best fit for your research. If you are not sure, read the appropriate sections before making your selection.

☒ Life sciences ☐ Behavioural & social sciences ☐ Ecological, evolutionary & environmental sciences

For a reference copy of the document with all sections, see nature.com/documents/nr-reporting-summary-flat.pdf

Life sciences study design

All studies must disclose on these points even when the disclosure is negative.

Sample size	the cell concentration was determined using hemocytometer, followed by normalization where applicable, as indicated in the manuscript
Data exclusions	no data were excluded from the analysis
Replication	Biological triplicates were analyzed to confirm data reproducibility. That is, the growth was conducted and experimental conditions were applied on separate cultures, cells were normalized and analyzed using basic statistical methods.
Randomization	not applicable, the cell number of a given strain (indicated in the manuscript) assures randomization
Blinding	not applicable, the cell number of a given strain (indicated in the manuscript) assures blinding

Reporting for specific materials, systems and methods

We require information from authors about some types of materials, experimental systems and methods used in many studies. Here, indicate whether each material, system or method listed is relevant to your study. If you are not sure if a list item applies to your research, read the appropriate section before selecting a response.

Materials & experimental systems

n/a	Involved in the study	
☐	☒	Antibodies
☐	☒	Eukaryotic cell lines
☒	☐	Palaeontology and archaeology
☒	☐	Animals and other organisms
☒	☐	Clinical data
☒	☐	Dual use research of concern

Methods

n/a	Involved in the study	
☒	☐	ChIP-seq
☒	☐	Flow cytometry
☒	☐	MRI-based neuroimaging

Antibodies

Antibodies used	TSPP1 was detected using antipeptide serum raised against C. reinhardtii TSPP1. GPX5 was detected with a commercially available antibody (AS15 2882, dilution 1:1000) obtained from Agrisera (Vännäs, Sweden). CHLI1 antibody (PHY5510S, dilution 1:1000) was purchased from PhytoAB (San Jose, CA, USA).
Validation	TSPP1 antibody was commercially validated using Elisa titers against the antigen. GPX5 and CHLI1 antibody are commercially available, as indicated in the Methods section.

Eukaryotic cell lines

Policy information about cell lines and Sex and Gender in Research

Cell line source(s)	Three strains used in this study were described elsewhere, chlD-1 in von Gromoff, et al. (2008), chlD-1/GUN4 in Brzezowski, et al. (2014), and wild type (4A+) in Dent, et al. (2005).
Authentication	Strains were authenticated in respective publications.
Mycoplasma contamination	Cell lines were not tested for mycoplasma contamination
Commonly misidentified lines (See ICLAC register)	not applicable
